# Gastric mucosal repair by Men’s Huwei Powder via EGF-NO/PGE2-PI3K-TLR4 in RELISH: Restoring Equilibrium through long-term integration of synergistic health

**DOI:** 10.3389/fphar.2025.1594089

**Published:** 2025-07-11

**Authors:** Kaijie Xu, Jing Li, Shoupeng Guo, Jin Zhang, Xichun Zhang

**Affiliations:** ^1^ College of Chinese Materia Medica and Food Engineering, Shanxi University of Chinese Medicine, Yuci, China; ^2^ College of Veterinary Medicine, Shanxi Agricultural University, Taigu, China

**Keywords:** gastric mucosal injury (GMI), RELISH framework, EGF-NO/PGE2, PI3K/Akt/NF-κB, TLR4/MyD88/NF-κB, gut microbiota, serum metabolite

## Abstract

**Background:**

Gastric mucosal injury (GMI) involves inflammation, oxidative stress, and barrier dysfunction. Existing therapies offer limited efficacy with side effects. Men's Huwei Powder (MHWP), a Traditional Chinese Medicine (TCM) formula developed under the RELISH (Restoring Equilibrium through Long-term Integration of Synergistic Health) framework, aims to restore mucosal and systemic equilibrium.

**Methods:**

The experiment was grouped using a uniform design, followed by the construction of an ethanol-induced GMI rat model. The effects of MHWP were assessed through histological examination, ELISA, RT-PCR, 16S rRNA sequencing, and LC-MS-based serum fingerprint analysis. Multivariate modeling techniques, including SPRA, PLSR, and pSEM, were utilized to explore the comprehensive regulatory mechanisms of MHWP.

**Results:**

MHWP promotes activation of the EGF–NO/PGE2 axis and concurrently suppresses key nodes of the PI3K/Akt/NF-κB signaling pathway, leading to downregulation of pro-inflammatory cytokines—including IL-1β, IL-6, and TNF-α—in both serum and gastric tissue. Beyond its localized effects, MHWP exerts systemic benefits by inhibiting hepatic TLR4, MyD88, and NF-κB expression, thereby reducing liver-derived IL-6 and TNF-α and ameliorating ethanol-induced liver injury. This hepatic protection contributes to improved gastric mucosal healing and systemic inflammatory balance. Gut microbiota profiling identified key genera—such as *Ligilactobacillus, Acutalibacter*, and *Lachnospiraceae*:CAG_95—as critical mediators of mucosal repair, with MHWP modulating their abundance in a botanicals–dependent manner. These genera were closely linked to the regulation of NO, COX-2, and gastric IL-1β and IL-6, highlighting their critical role in the EGF–NO/PGE2 axis and inflammatory signaling. Serum HPLC fingerprinting identified several bioactive metabolites—including 6-gingerol (P1), atractylenolide II (P10), and dihydrostilbene base + 3O,2Prenyl (P18)—as major contributors to MHWP's efficacy. Closely associated with specific botanical drugs, these metabolites synergistically regulated multiple inflammatory and reparative pathways, underscoring MHWP's holistic therapeutic mechanism.

**Conclusion:**

MHWP exerts multi-targeted effects through integrated modulation of the liver, gut microbiota, and serum metabolites. These findings underscore its potential as a holistic and sustainable TCM-based intervention for GMI, in alignment with the RELISH framework.

## 1 Introduction

Gastric mucosal injury (GMI), a widespread condition characterized by the disruption of the stomach’s protective lining, poses significant clinical challenges due to its multifactorial etiology and complex pathophysiology (Lanas and Chan, 2017). Conventional treatments often focus on alleviating symptoms or neutralizing gastric acid, while neglecting the interconnected molecular pathways essential for effective mucosal repair (Oshima and Miwa, 2016). Emerging research highlights the need for a holistic, multi-targeted therapeutic approach that not only addresses the underlying causes of GMI but also promotes comprehensive healing by restoring homeostasis across physiological systems (Chen et al., 2023).

At the heart of this holistic approach is the traditional Chinese medicine (TCM) principle of “*Da-Bing-Yi-Wei*”, introduced by Prof. Men Jiu-Zhang (Men, 2016), which emphasizes the central role of gastrointestinal health in preventing and treating severe diseases. This principle proposes that the gastrointestinal system is not only responsible for digestion but also plays a fundamental role in maintaining systemic health by balancing internal energies. *Wei-Qi*, a critical TCM concept in this principle, represents the digestive and immune functions that protect the body and restore balance (Zhang et al., 2025). Specifically, *Wei-Qi* helps to fortify the body’s defenses, enhance digestion, and support immune function, making it a cornerstone of GMI treatment. In the context of GMI, strengthening *Wei-Qi* helps to restore the integrity of the gastric mucosa and modulate the immune response, promoting healing and preventing further damage (Fu and Yang, 2019).

Building upon this foundation, the Restoring Equilibrium through Long-term Integration of Synergistic Health (RELISH) framework incorporates these principles into a structured strategy that addresses the limitations of conventional medicine. By emphasizing a comprehensive understanding of health and disease, the RELISH framework promotes the proactive restoration of *Wei-Qi*, which is central to achieving holistic recovery. This approach integrates dietary, lifestyle, and botanical drug strategies, enabling patients to actively engage in their healing process and foster long-term well-being. Incorporating both traditional principles and modern medical insights, the RELISH framework underscores the gastrointestinal system’s crucial role in overall health, highlighting the interconnected functions of gastrointestinal health, immunity, and tissue repair.

Specifically, in the context of GMI repair, the RELISH framework emphasizes three interconnected pillars: epithelial regeneration, inflammation modulation, and vascular restoration. Epithelial regeneration is primarily mediated by the epidermal growth factor (EGF) and its receptor (EGFR), stimulating cellular proliferation and migration to restore the gastric lining through precise signaling pathways (Shimamoto et al., 2003; Tarnawski and Ahluwalia, 2021). Complementing this is the enhancement of Nitric Oxide (NO) and prostaglandin E2 (PGE2), which synergistically improve microvascular perfusion and promote mucosal protection (Lanas, 2008; Marmol et al., 2009). Meanwhile, the suppression of pro-inflammatory signaling through pathways such as PI3K/Akt/NF-κB ensures a balanced immune response, preventing excessive tissue damage while facilitating effective repair (Liu et al., 2017; Saravia et al., 2020). These mechanisms underscore the efficacy of multi-targeted botanical interventions in achieving holistic gastric mucosal repair.

Building on this biomedical foundation, the roles of liver function and gut microbiota further enrich the RELISH framework, highlighting their complementary contribution to both localized gastric mucosal repair and systemic homeostasis. In biomedicine, hepatic regulation of systemic inflammation—particularly through the TLR4/MyD88/NF-κB axis—plays a crucial role in mucosal repair by modulating cytokine profiles and systemic immune responses (Thiel et al., 2023; Song et al., 2024). Meanwhile, the gut microbiota, as a key modulator of gastrointestinal health, interacts dynamically with botanical formulations to enhance pharmacological efficacy (Yue et al., 2021). Specific bacterial genera, such as *Roseburia* and *Lactobacillus*, have been identified as important mediators of mucosal healing, underscoring the value of microbiota-driven therapeutic pathways (Feng et al., 2023; Ekstedt et al., 2024). These modern insights align closely with the TCM theory of *Gan-Pi* Regulation, which emphasizes the interconnected roles of *Gan* and *Pi* in maintaining systemic balance and digestive health. In TCM, Gan governs the regulation of *Qi*—the vital energy that sustains physiological functions—and stores *Xue*, the nourishing essence that supports organ activity and internal harmony. *Pi*, responsible for transforming food into *Qi* and *Xue*, provides essential energy and nourishment. Its digestive and transformative function parallels the role of gut microbiota in nutrient absorption, metabolic regulation, and immune modulation. The coordinated actions of *Gan* and *Pi* ensure the smooth flow of *Qi* and *Xue*, a concept that mirrors the dynamic interplay between hepatic function and gut microbiota in promoting digestive health and systemic homeostasis. Together, these perspectives illustrate a convergent understanding: both TCM and biomedical science recognize the liver and gut microbiota as complementary agents in sustaining gastrointestinal integrity and overall vitality. This integrative view is foundational to the RELISH framework’s multi-targeted therapeutic model, which addresses not only localized gastric mucosal repair but also broader systemic regulation and balance.

A key feature of the RELISH approach is its focus on multi-targeted interventions that utilize the synergistic effects of botanical drugs. Unlike single-target pharmacological therapies, this strategy leverages their natural compatibility to activate protective pathways while inhibiting harmful processes (Ospondpant et al., 2024). Men’s Huwei Powder (MHWP), developed by Professor Men Jiu-Zhang, exemplifies this principle with its innovative design and proven efficacy. Rooted in the traditional *Li Zhong decoction*, as recorded in the Chinese medical classic *Shang Han Lun*, MHWP reflects decades of clinical experience in treating gastrointestinal disorders (Men, 2016; Song et al., 2020). MHWP incorporates a hierarchical blend of botanical drugs specifically designed to target multiple pharmacological niches of mucosal repair, exemplifying TCM’s integrative and multi-targeted approach (Details of the botanical drugs in MHWP are provided in Supplementary Table S1.

The integration of active serum metabolites from botanical metabolites into the RELISH framework provides a molecular basis for its multi-targeted efficacy. 6-Gingerol and Atractylenolide II, associated with *Zingiberis Rhizoma* (ZR) and *Largehead Atractylodes Rhizome* (LAR) respectively, have been shown to support tissue repair and systemic anti-inflammatory effects (Liu et al., 2020; Xie et al., 2023). As key botanical drugs in MHWP, ZR and LAR illustrate this formula’s multi-targeted approach, integrating these effects to advance its holistic strategy for gastric mucosal repair. This biochemical complexity underscores the potential of the RELISH framework to harmonize modern molecular pharmacology with TCM’s holistic principles.

Therefore, it is hypothesized that MHWP promotes gastric mucosal repair through multi-target mechanisms, including the PI3K/Akt/NF-κB pathway and EGF-NO/PGE2 axis, supporting epithelial regeneration, inflammation modulation, and vascular restoration. Furthermore, liver function and inflammation mediated by TLR4/MyD88/NF-κB, along with intestinal flora, are also believed to play critical roles in this process. Additionally, serum metabolites derived from botanical drugs are thought to underpin its multi-targeted efficacy.

Here, this study integrates a uniform design (UD) and advanced analytical methods to optimize experimental efficiency and enhance mechanistic insights. To explore the hierarchical structure of botanical drugs in MHWP, this study utilized Least Absolute Shrinkage and Selection Operator (LASSO) regression combined with Stepwise Polynomial Regression Analysis (SPRA) to analyze the relationship between botanical drugs and efficacy indicators. Additionally, Spearman correlation analysis, Partial Least Squares Regression (PLSR), and piecewise Structural Equation Modeling (pSEM) were employed to investigate the roles of liver function, gut microbiota, and serum metabolites in gastric mucosal protection. Collectively, these methodologies underscore the RELISH approach of MHWP, which employs an integrative and holistic strategy to achieve multi-targeted gastric mucosal repair.

## 2 Materials and methods

### 2.1 Materials and reagents

Paraformaldehyde (Lot No. 1340040101602, ≥98% purity) was procured from Sichuan Xilong Scientific Co., Ltd. (Chongqing, China). Absolute ethanol (Lot No. 100092680, ≥98% purity), methanol (Lot No. 10014118, ≥98% purity), and acetonitrile (Lot No. 40064160, ≥98% purity) were supplied by Sinopharm Chemical Reagent Co., Ltd. (Shanghai, China). Hematoxylin (Lot No. G1004) and eosin (Lot No. YE2080) staining solutions were obtained from Servicebio Technology Co., Ltd. (Wuhan, China) and Bomei Biotechnology Co., Ltd. (Hefei, China), respectively.

### 2.2 Uniform design and preparation of TCM granules

Uniform Design (UD) was employed to determine the hierarchical structure of botanical drugs in MHWP. UD is an experimental methodology that optimizes experimental efficiency by ensuring uniform coverage of the experimental space while minimizing the number of trials required (Joseph, 2016). This approach is particularly valuable for optimizing complex formulations like MHWP, where balancing multiple variables is essential for effective treatment outcomes. Supplementary Table S2 presents the formulations derived from the MHWP formula using the U_7_ (7^6^) Uniform Design table, generated with the Data Processing System (DPS) software (Tang and Zhang, 2013). The botanical drug grammages were adjusted within reasonable dosage ranges as specified in the “Pharmacopoeia of the People’s Republic of China” (Committee, 2020). All botanical materials were procured from Beijing Tongrentang Co., Ltd. (Beijing, China), authenticated by the College of Chinese Materia Medica and Food Engineering, Shanxi University of Chinese Medicine, and verified to meet the standards outlined in the Pharmacopoeia of the People’s Republic of China (2020 Edition).

MHWP comprises six traditional Chinese botanical ingredients with the following quantities: Zingiberis Rhizoma (ZR) 4 g, Glycyrrhizae Radix et Rhizoma (GCR) 6 g, Largehead Atractylodes Rhizome (LAR) 9 g, Codonopsis Radix (CR) 6 g, Forsythia Fruit (FF) 4 g, and Pinelliae Rhizoma Praeparatum Cum Zingibere Et Alumine (PR) 6 g. Powders for each experimental formulation were prepared using quintuple formulas under standardized procedures. The botanical ingredients were ground into fine powders and passed through a 100-mesh sieve, with the finer fractions set aside. The coarser material, along with the remaining unprocessed botanical ingredients, was soaked in 4 L of purified water for 1 h. Following this, the mixture was boiled for 1 h to extract bioactive metabolites. The concentrated extracts were then blended with the reserved fine powders using a high-shear mixer, where wet granulation was performed. The resulting granules were sterilized and dried at 65°C for 30 min to produce the final preparation.

### 2.3 Animals and their treatment

Male Sprague-Dawley rats (210 ± 30 g, 6–8 weeks old) were obtained from Yi Fengda Biotechnology Co., Ltd. (Xi’an, China; Certificate No.: SYXK (Shan) 2020–006). The animals were housed under standardized environmental conditions (23°C ± 2°C, 55%–65% relative humidity, with adequate ventilation) and were acclimatized for 7 days with unrestricted access to water and standard laboratory chow prior to the experiment. All experimental procedures were reviewed and approved by the Institutional Animal Care and Use Committee (IACUC) of the College of Veterinary Medicine, Shanxi Agricultural University. Efforts were made to minimize animal discomfort and pain during all experimental procedures, including surgeries, in accordance with ethical guidelines for animal research.

Rats were randomly assigned to 10 groups (n = 5 per group), including a control group, a model group, an MHWP group, and seven Uniform Design (UD1–UD7) groups. Based on clinical guidelines, the recommended daily dose of MHWP for a 70-kg adult is 35 g of crude botanical drugs, which undergo a standardized technical process to yield the final medicinal form of MHWP, corresponding to approximately 0.5 g/kg/day. For preclinical studies in rats, a body surface area (BSA)-based conversion factor of 6.3 was applied, following standard interspecies scaling protocols (Nair et al., 2018). This resulted in a rat-equivalent dose of 3.15 mg/g/day, calculated by multiplying the human dose (0.664 mg/g) by the conversion factor.

The daily doses of MHWP and UD1–UD7 formulations were prepared in warm purified water and adjusted to an administration volume of 10 mL/kg to ensure consistent dosing across groups. Throughout the experiment, rats had free access to basic diet and water, except for a 12-h fasting period prior to ethanol administration to ensure gastric emptying and maximize ethanol contact with the gastric mucosa for successful model induction. MHWP solutions were administered by oral gavage once daily for 3 days. On the fourth day, the control group received 8 mL/kg of normal saline, while all other groups were administered 8 mL/kg of 75% ethanol to induce gastric mucosal injury (GMI). Treatment with MHWP or the UD1–UD7 formulations continued for three additional days post-induction. One hour after the final gavage, blood samples were collected via the tail vein and centrifuged at 3,000 rpm for 15 min to isolate serum. Meanwhile, fecal samples were collected in 2 mL enzyme-free tubes and stored at −80°C for subsequent 16S rRNA sequencing. Subsequently, all rats were euthanized to collect gastric and liver tissue samples for further analysis.

### 2.4 HPLC-MS analysis

200 μL of serum was combined with 400 μL of methanol, vortex-mixed for 5 min, and centrifuged at 12,000 rpm for 15 min at 4°C. The resulting supernatant was collected, filtered through a 0.22 μm membrane, and transferred into liquid chromatography vials for high-performance liquid chromatography (HPLC) analysis. Chromatographic separation was performed using an Agilent 1,260 Infinity Ⅱ system equipped with an SB-C18 column (4.6 mm × 250 mm, 5 μm, Agilent Technologies, China) maintained at 28°C. The mobile phase consisted of 0.1% formic acid in water (Phase A) and 0.1% formic acid in acetonitrile (Phase B), delivered at a flow rate of 0.4 mL/min. The injection volume was set to 20 μL. The gradient elution program was as follows: 0–3.5 min, 95% Phase A; 3.5–6.5 min, 70% Phase A; 6.6–12.5 min, 30% Phase A; 12.5–25 min, 0% Phase A; and 25–30 min, 95% Phase A. UV detection was performed at a wavelength of 254 nm.

Chromatographic fingerprints were analyzed using the “Traditional Chinese Medicine Chromatographic Fingerprint Similarity Evaluation System” (Li et al., 2024). Chromatographic peaks from serum samples derived from MHWP-treated rats and UD1-UD7 groups were compared with those from model rats. Peaks with identical retention times were excluded from the analysis.

Mass spectrometry (MS) analysis was conducted using a Thermo Scientific Q Exactive high-resolution liquid chromatography-mass spectrometry system (Thermo Fisher Scientific, United States). The electrospray ionization (ESI) interface was operated in both positive and negative ionization modes under the following conditions: capillary temperature at 350°C, spray voltage at 3.0 kV, capillary voltage at 20 V, sheath gas (N2) flow rate of 40 arb, and auxiliary gas (N2) flow rate of 15 arb. Full-scan data acquisition was carried out in MS scan mode, covering an m/z range of 100–1,000, with collision energy values automatically adjusted to optimize ion fragmentation.

Subsequent MS data processing was performed using MZmine 2, following a standardized workflow that included raw data import, peak detection, and adduct deconvolution. Metabolites were identified by evaluating the retention time (Rt) deviation relative to reference materials, precursor ion mass accuracy, matching of fragment ion spectra, and isotopic distribution patterns. The processed parameters were matched against a custom-built database, curated from theoretical datasets derived from published literature and publicly available resources.

### 2.5 Macroscopic examination

Gastric tissue samples were dissected to visually assess mucosal injury. The length, width, and number of hemorrhagic lesions were measured using an electronic caliper for quantitative analysis. Gross pathological evaluation of gastric mucosal damage was performed using a validated 0–5 scoring system based on lesion number and severity, as previously described (Arab et al., 2015). The scoring criteria were as follows: 0 = no lesions; 1 = small, round hemorrhagic lesions; 2 = lesions <2 mm; 3 = lesions 2–3 mm; 4 = lesions 3–4 mm; 5 = lesions >4 mm. For lesions with an erosion width exceeding 1 mm, the score was multiplied by 2 to account for the increased severity. The ulcer index was calculated as the sum of the individual lesion scores, providing a comprehensive measure of mucosal damage severity.

### 2.6 Histology assay

Gastric tissue samples were fixed in 10% neutral buffered formalin for 24 h, dehydrated through a graded ethanol series, and embedded in paraffin. Five-micron-thick sections of the fundic mucosa were obtained from each paraffin block, and serial sections were stained with hematoxylin and eosin (H&E) for histopathological analysis. Micropathological damage was assessed by a certified pathologist who was blinded to the treatment groups, following a previously validated methodology (Laine and Weinstein, 1988).

### 2.7 Serum liver function biochemical assay

Serum ALT, AST, and TBIL levels were measured using specific assay kits, all purchased from Dirui Medical Technology Co., Ltd. (Changchun, China).

### 2.8 Enzyme-linked immunosorbent assay (ELISA)

The concentrations of various pharmacological indicators in rat stomach and serum samples were measured using ELISA kits for NO, Enos, PGE2, EGF, EGFR, COX-2, IL-1β, IL-6, TNF-α, NF-κB, Akt, PI3K, MyD88, and TLR4 from Meimian Industrial Co., Ltd. (Jiangsu, China), following the manufacturer’s protocols.

### 2.9 Quantitative real-time PCR analysis

Total RNA was extracted using the mirVana RNA Isolation Kit (Thermo Scientific, United States), and cDNA synthesis was performed with the TransScript All-in-one SuperMix for qPCR according to the manufacturer’s instructions. Real-time PCR was conducted using PerfectStart Green qPCR SuperMix, following the protocol provided by TransGen Biotech Co., Ltd. (Beijing, China). Primers were synthesized by Sangon Biotech Co., Ltd. (Shanghai, China) with the following sequences: rat actin (forward 5′-GCG​AGT​ACA​ACC​TTC​TTG​C-3′, reverse 5′-TAT​CGT​CAT​CCA​TGG​CGA​AC-3′); rat eNOS (forward 5′-GTG​TCC​AAT​ATG​CTG​CTA​GAA-3′, reverse 5′-TAT​CGT​CAT​CCA​TGG​CGA​AC-3′); rat COX-2 (forward 5′-TTC​CTC​CTG​TGG​CTG​ATG​ACT​GC-3′, reverse 5′-GTG​CTG​GGC​AAA​GAA​TGC​GAA​C-3′); rat EGF (forward 5′-CGG​ATT​AAC​ACA​GAT​GGA​AC-3′, reverse 5′-CCC​AAT​AGA​GTC​TCT​CTT​CCT-3′); and rat EGFR (forward 5′-TCT​GTG​CAG​AAC​CCA​GTC-3′, reverse 5′-CTG​GGC​AGT​GTT​GAG​ATA​C-3′); rat PI3K (forward 5′-AAG​TTC​AGC​GAG​GAC​AAG​ATT​CAG-3′, reverse 5′-GTT​CTC​CAG​GAC​GGA​CGG​TTC-3′); rat Akt (forward 5′-ATC​GTG​TGG​CAA​GAT​GTG​TAT​GAG-3′, reverse 5′-GCT​GAG​TAG​GAG​AAC​TGG​GGA​AA-3′); rat NF-κB (forward 5′-GAG​ACC​TGG​AGC​AAG​CCA​TTA​GC-3′, reverse 5′-AGT​GTT​GGG​GGC​ACG​GTT​A-3′); rat TLR4 (forward 5′-CAT​TGT​TCC​TTT​CCT​GCC​TGA​G-3′, reverse 5′-TAG​GTT​CTT​GGT​TGA​ATA​AGG​GAT​G-3′); rat MyD88 (forward 5′-AGG​ACT​GCC​AGA​AAT​ACA​TAC​GC-3′, reverse 5′-ATG​CCT​CCC​AGT​TCC​TTT​GTC-3′).

### 2.10 16S rRNA sequencing

16S ribosomal DNA sequencing of fecal samples was performed by Personalbio Biotechnology Co., Ltd. (Shanghai, China). Detailed methods are provided in the Supplementary Material (Supplementary Method).

### 2.11 Statistical analysis

Significance testing was performed using SPSS 26.0. One-way ANOVA followed by Tukey’s HSD test was applied for comparisons when equal variances were assumed, and the Games-Howell test was used for cases of unequal variances. Composite indices were calculated using Equations 1, 2, with the weight coefficients determined through the EWM (Huang et al., 2021).

For the UD experiments, data analysis was performed using R version 4.4.1. The first step in analyzing the botanicals-effect relationship involved using LASSO regression to reduce dimensionality and identify potential predictors. LASSO regression eliminates non-significant factors, helping to prevent overfitting and simplifying high-dimensional data (Tibshirani, 2011). In this process, the predictors were the polynomial effects of botanical drugs, including linear, quadratic, and interaction terms, which represent the main effects of individual botanical drugs, their nonlinear effects, and their interactions, respectively. The response variable was the mean of the pharmacological indicators for each UD formulation. Following this, the SPRA was applied to further refine the selection of variables. This iterative approach further filters out redundant predictors, improving model accuracy (Peres and Fogliatto, 2018). Specifically, the “step” function (with the parameter direction = “both”) was used to iteratively refine the model, starting with the botanical drugs selected by LASSO. The performance of the SPRA model was evaluated based on R^2 values and p-values to ensure robust predictor selection.

Spearman correlation analysis was used to examine the relationship between liver markers and indicators associated with GMI amelioration. PLSR was employed to examine the relationships between fingerprint profiles and gut microbiota composition with pharmacological indicators separately. This method extracts informative latent variables and screens biomarkers, making it well-suited for handling complex multivariate data (Yu et al., 2024). To optimize the models, the number of components (ncomp) was set to 2 for the microbiota-effect PLSR model and 3 for the fingerprint-effect PLSR model, based on the criterion that the cumulative explained variance of the dependent variable (Y) exceeded 80%. In the gut microbiota-pharmacological effect PLSR analysis, the predictors were the abundances of bacterial genera identified by the LESef analysis. These genera represented the differences between the control and model groups, as well as between the model and MHWP groups, with duplicate taxa removed and those present in only a small number of samples excluded. For the fingerprint-pharmacological effect PLSR, the predictors were the peak areas of the characteristic peaks. Variables with Variable Importance in Projection (VIP) scores greater than 1 were identified as significant contributors. Additionally, normalization across diverse pharmacological indices was performed using the Z-score standardization method to ensure comparability across datasets (Gong et al., 2021). The “pSEM” was used for path analysis (Klein-Raufhake et al., 2025). In constructing the pSEM model, predictor variables from SPRA and mediating variables with VIP scores above 1 from PLSR analysis formed the initial model. This model was iteratively refined by removing non-significant pathways, and adequacy was evaluated using the Fisher’s C statistic until the final model was obtained (Lefcheck, 2016). Model fit was further assessed using the Akaike Information Criterion (AIC).

$$Y=\sum_{j}\left(\frac{W_{j}-Wmin}{W\max-Wmin}\times E_{j}\right)$$ (1)

$$Y=\sum_{j}\left(\frac{W_{j}-Wmin}{W\max-Wmin}\times E_{j}\right)$$ (2)

Where *Y* denotes the composite index, *Wj* is the value of the *j*-th indicator, *Wmax* and *Wmin* represent the maximum and minimum values of the *j*-th indicator within the dataset, respectively, and *Ej* is the entropy weight coefficient of the *j*-th indicator. Equation 1 is used for positive indicators, where higher values indicate better outcomes, while Equation 2 is applied to negative indicators, where lower values are more favorable.

## 3 Results

### 3.1 Multi-targeted approach of MHWP with hierarchical formulation on GMI

#### 3.1.1 Gastric mucosal injury and pathophysiology

**FIGURE 1:**
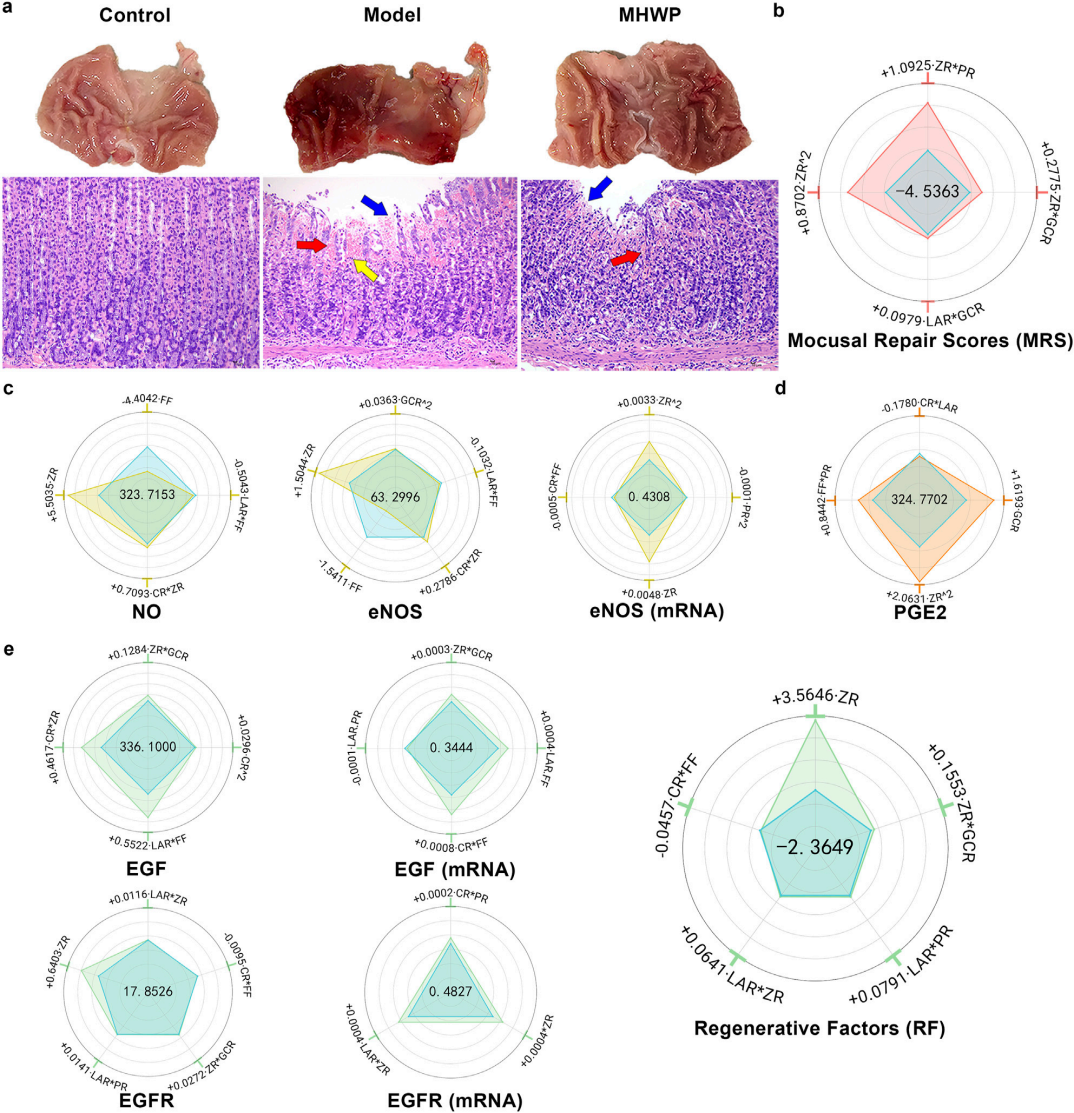
Hierarchical botanical drugs in MHWP promoted gastric mucosal repair by regulating the EGF-NO/PGE2 pathway. **(a)**, Morphology of gastric mucosa and histology of gastric mucosa (H&E staining). Red arrows indicate red blood cell exudation, yellow arrows indicate inflammatory cell infiltration, and blue arrows indicate mucosal cell damage and loss. **(b)** Radar chart for SPRA of Y_MRS_. The area formed by the blue dots indicates the intercept, visually represented at the center of the radar plot. The areas formed by dots of other colors correspond to the standardized regression coefficients, illustrating both the sign (positive or negative) and the relative magnitude of each variable’s contribution. The dots representing the coefficients within the blue reference area correspond to negative effects, whereas those positioned outside this area indicate positive effects. This graphical convention is consistently applied across all relevant figures in this study. **(c)**, Radar chart for SPRA of Y_NO,_ Y_eNOS,_ and Y _eNOS(mRNA)_. **(d)**, Radar chart for SPRA of Y_PGE2_. **(e)**, Radar chart for SPRA of Y_EGF,_ Y_EGF(mRNA),_ Y_EGFR,_ Y_EGFR(mRNA)_ and Y_RF_.

MHWP significantly mitigated ethanol-induced gastric bleeding, edema, mucosal shedding, and inflammatory cell infiltration in rats (Figure 1a). These therapeutic effects were evidenced by substantial reductions in both the Ulcer Index (UI) and Histopathological Scores (HS), highlighting its potent restorative effects on GMI (Supplementary Table S3).

Y_MRS_ (Mucosal Repair Scores), a composite index integrating UI and HS with respective weight coefficients of 0.5269 and 0.4731 determined through the Entropy Weight Method (EWM), provided a quantitative measure of overall repair efficacy. Indicated by the regression equation of Y_MRS_ derived from SPRA, the results showed that the CR and FF had no significant effect on Y_MRS_, while ZR^2 and ZR*PR had the most primary positive effects, underscoring the critical roles of botanical drugs ZR and PR in promoting gastric mucosal repair. Additionally, ZR*GCR and LAR*GCR had secondary positive effects on Y_MRS_, indicating the supportive roles of botanical drugs GCR and LAR in the repair process alongside the critical botanical drugs (Figure 1b).

#### 3.1.2 Serum NO and eNOS expression in gastric tissue

MHWP significantly elevated serum NO levels and enhanced both protein and mRNA expression of eNOS in gastric tissue (Supplementary Table S4).

ZR exhibited the most substantial positive influence on Y_NO_, Y_eNOS_, and Y_eNOS(mRNA)_, with ZR^2 exhibiting a significant association with increased Y_eNOS(mRNA)_. These results highlight ZR as a pivotal botanical drug in augmenting serum NO levels. Furthermore, ZR*CR demonstrated a minor positive contribution to Y_NO_ and Y_eNOS_, and GCR^2 exerted a moderate positive effect on Y_eNOS_, underscoring that the botanical drugs CR and GCR play complementary roles in enhancing NO production within the framework of MHWP formulation. In contrast, FF displayed a pronounced negative effect on Y_NO_ and Y_eNOS_, accompanied by FF*LAR marginally reducing these levels and FF*CR causing a minor decrease in Y_eNOS (mRNA)_. These findings indicate that the botanical drug FF may act as a limiting factor for NO synthesis. Also exerting a minor negative influence, PR^2 downregulated Y_eNOS(mRNA)_ (Figure 1c).

#### 3.1.3 PGE2 concentrations in gastric tissue

MHWP effectively counteracted ethanol-induced PGE2 reduction (Supplementary Table S5). Further regression analysis revealed that ZR^2 significantly enhanced Y_PGE2_, establishing it as a pivotal botanical drug in MHWP for promoting mucosal repair. Additionally, GCR^2 secondarily enhanced Y_PGE2_, while FF*PR slightly increased Y_PGE2_, indicating complementary roles of GCR, FF, and PR in supporting PGE2 production. In contrast, CR*LAR slightly reduced Y_PGE2_, suggesting potential antagonistic interactions in the formulation (Figure 1d).

#### 3.1.4 EGF and EGFR protein and mRNA expression in gastric tissue

MHWP significantly enhanced the protein and mRNA expression levels of EGF and EGFR in gastric tissue (Supplementary Table S6).

FF*LAR significantly promoted both Y_EGF_ and Y_EGF(mRNA)_, with its effect on Y_EGF_ being the most pronounced. Meanwhile, FF*CR exhibited the strongest upregulated influence on Y_EGF (mRNA)_. These findings underscore the critical role of the botanical drug FF in modulating EGF expression in gastric tissue, with LAR and CR synergistically interacting to enhance EGF expression. Furthermore, ZR*CR had a secondary positive effect on Y_EGF_, while ZR*GCR exhibited a slight positive effect on both Y_EGF_ and Y_EGF (mRNA)_. These findings suggest that the botanical drug ZR plays a supportive role in promoting EGF expression through synergistic regulation with the botanical drugs GCR and CR. Conversely, LAR*PR exerted a slight negative effect on Y_EGF (mRNA)_, reflecting the complexity of this traditional botanical formula’s mechanisms and suggesting that its regulatory effects may vary due to dynamic interactions between the botanical drugs (Figure 1e).

ZR emerged as the most significant contributor to Y_EGFR_ and Y_EGFR (mRNA)_, underscoring its role as a key botanical drug in MHWP for enhancing EGFR production. Additionally, ZR*LAR exhibited a secondary positive effect on Y_EGFR_ and Y_EGFR (mRNA)_, accompanied by minor positive contributions from LAR*PR and ZR*GCR to Y_EGFR_, and CR*PR to Y_EGFR (mRNA)_. These findings highlight the roles of LAR, PR, and GCR as supportive botanical drugs that complement the critical effects of primary botanical drugs. Conversely, CR*FF showed a slight negative effect on Y_EGFR_ (Figure 1e).

Y_RF_ (Regenerative Factors), a composite index derived from Y_EGF_ and Y_EGFR_ with respective weight coefficients of 0.6304 and 0.3696, serves as a quantitative measure of gastric mucosal regeneration. ZR is the main contributor to the increase in RF, suggesting it is likely the key botanical drug responsible for the reparative effect of MHWP. Additionally, ZR*GCR and LAR*ZR show slight enhancing effects, indicating that GCR and LAR act as supportive botanical drugs through their interaction with ZR. Similarly, LAR*PR slightly enhances RF. In contrast, CR*FF has a minor negative effect on RF (Figure 1e).

#### 3.1.5 COX-2 expression in gastric tissue

**FIGURE 2:**
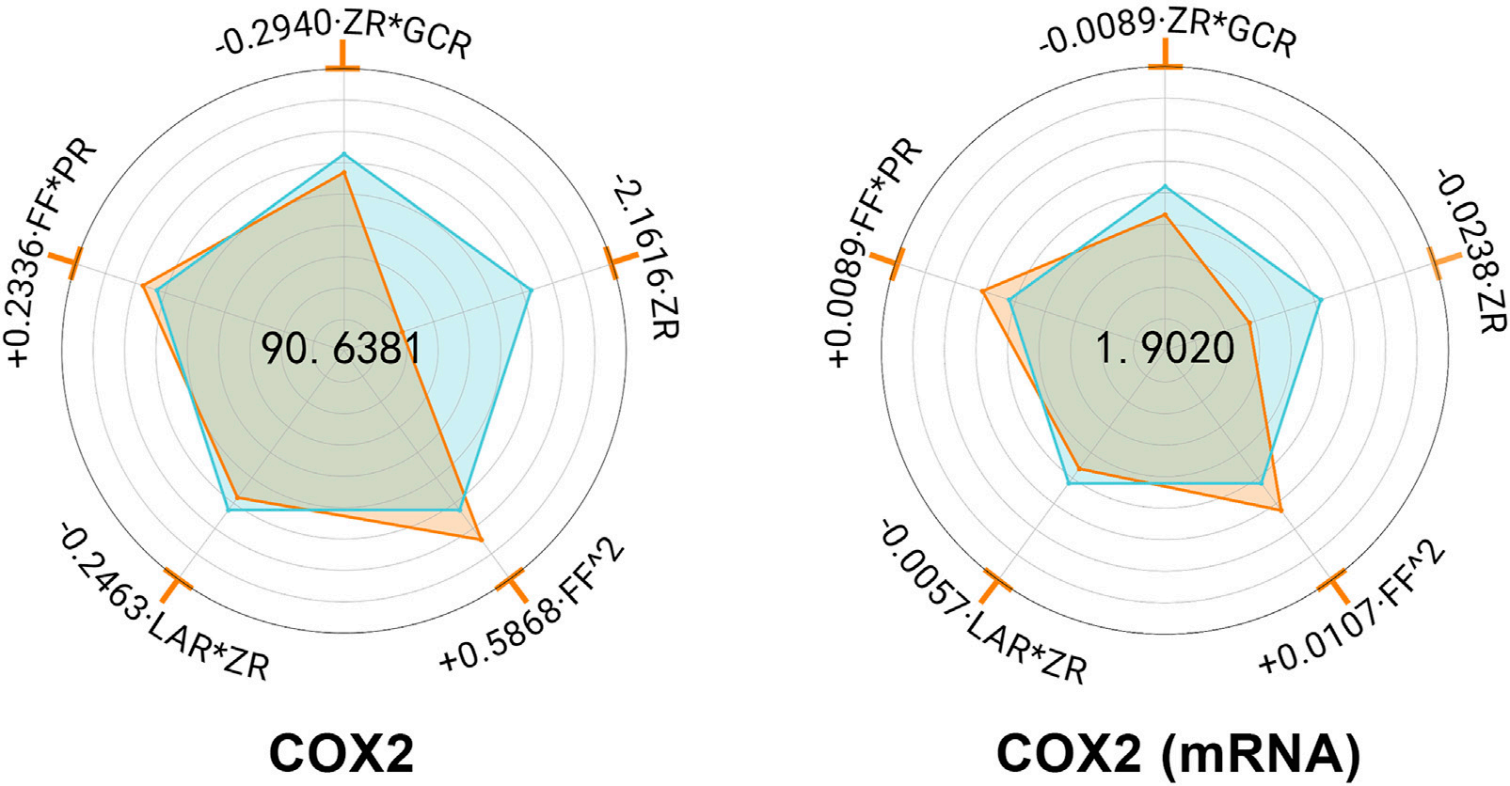
Hierarchical botanical drugs in MHWP regulated gastric COX-2 expression. Radar chart for SPRA of Y_COX-2 and_ Y_COX-2(mRNA)_.

MHWP significantly mitigated the ethanol-induced upregulation of COX-2 expression in gastric tissue (Supplementary Table S7). Further regression analysis, CR exhibited no significant effect, while ZR demonstrated the most pronounced downregulation, underscoring its pivotal role in reducing COX-2 expression. Additionally, ZR*GCR and ZR*LAR showed minor reductions in Y_COX-2_ and Y_COX-2 (mRNA)_, suggesting that GCR and LAR act as supporting botanical drugs in suppressing COX-2 expression alongside the primary botanical drugs. In contrast, FF^2 and FF*PR were associated with slight increases in Y_COX-2_ and Y_COX-2 (mRNA)_ expression levels (Figure 2).

#### 3.1.6 Inflammatory cytokines levels in gastric tissue and serum

MHWP markedly decreased the concentrations of pro-inflammatory cytokines IL-1β, IL-6, and TNF-α in both gastric tissue and serum, demonstrating its potent anti-inflammatory activity in protecting the gastric mucosa (Supplementary Table S8).

**FIGURE 3:**
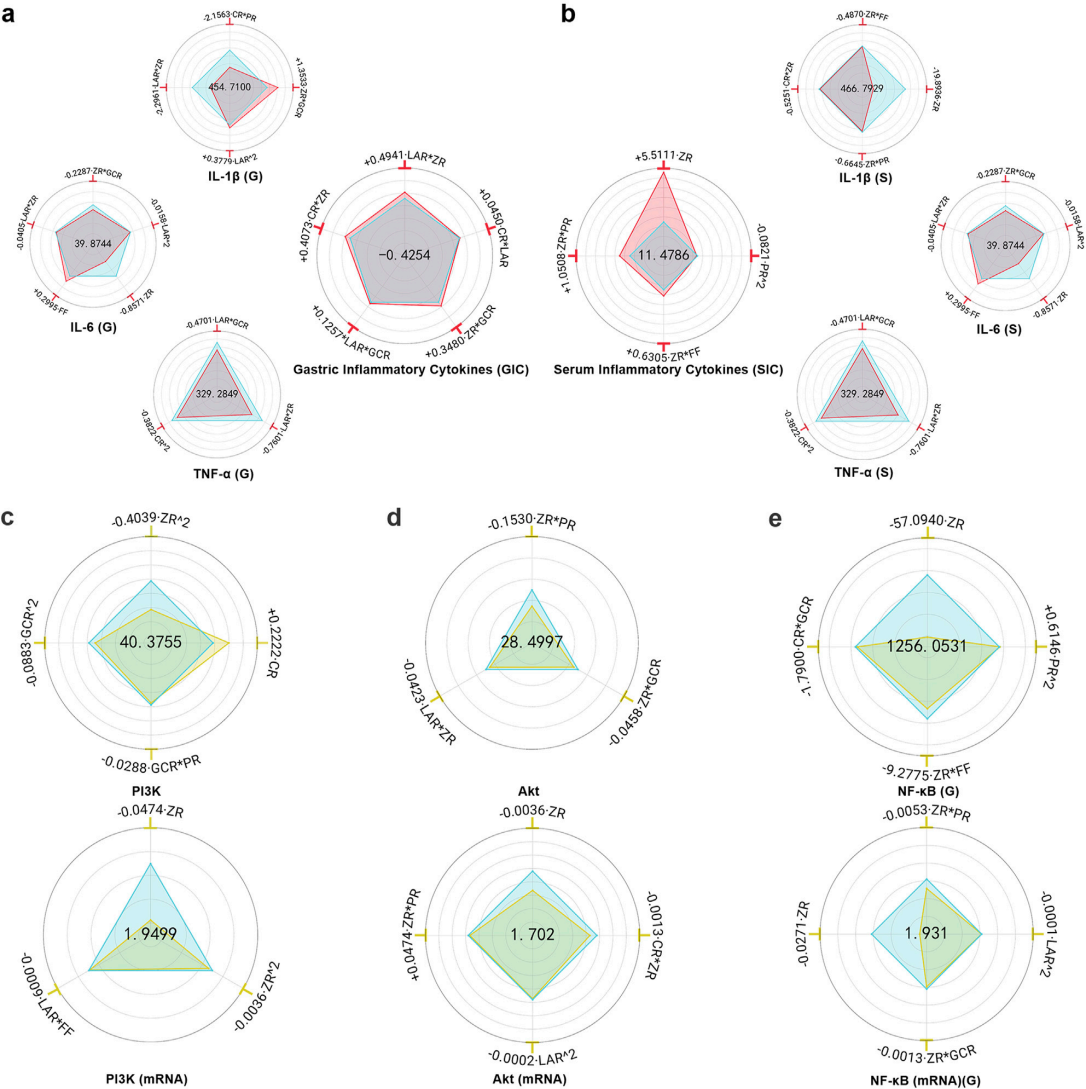
Hierarchical botanical drugs in MHWP regulated local and systemic inflammatory responses in gastric tissue via the PI3K/Akt/NF-κB pathway. **(a)**, Radar chart for SPRA of Y_IL-1β(G)_, Y_IL-6(G),_ Y_TNF-α(G)_ and Y_GIC_. **(b)**, Radar chart for SPRA of Y_IL-1β(S)_, Y_IL-6(S),_ Y_TNF-α(S)_ and Y_SIC_. **(c)**, Radar chart for SPRA of Y_PI3K_ and Y_PI3K(mRNA)_. **(d)**, Radar chart for SPRA of Y_Akt_ and Y_Akt(mRNA)_. **(e)**, Radar chart for SPRA of Y_NF-κB(G)_ and Y _NF-κB(mRNA) (G)_.

FF had no significant effect on gastric Y_IL-1β (G)_ or Y_TNF-α (G)_ and caused only a slight increase in Y_IL-6 (G)_, suggesting that the botanical drug FF unlikely play a major role in regulating inflammatory factors in gastric tissue. While ZR*LAR significantly reduced Y_IL-1β (G)_, Y_IL-6 (G)_, and Y_TNF-α (G)_, with ZR showing the strongest suppressive effect on Y_IL-6 (G)_. These findings identify ZR as a key botanical drug in MHWP for modulating gastric mucosal inflammation. Furthermore, LAR^2 and ZR*GCR exhibited minor suppressive effects on Y_IL-6 (G)_, while LAR*GCR and CR^2 showed slight reductions in Y_TNF-α (G)_. These results suggest that the botanical drugs LAR and GCR play supportive roles in regulating IL-6 and TNF-α levels in gastric tissue, with CR uniquely assists in modulating TNF-α levels. Notably, CR*PR demonstrated a significant reduction in Y_IL-1β(G)_, while PR had no significant effect on Y_IL-6 (G)_ or Y_TNF-α (G)_, indicating that the botanical drug PR primarily contributes to inflammation suppression by targeting Y_IL-1β (G)_ in gastric tissue. Conversely, LAR^2 and ZR*GCR showed a slight increase in Y_IL-1β (G)_ (Figure 3a).

Y_GIC_ (Gastric Inflammatory Cytokines), a composite index derived from Y_IL-1β (G)_, Y_IL-6 (G)_, and Y_TNF-α (G)_ with weight coefficients of 0.2373, 0.3023, and 0.4604, respectively, serves as a quantitative measure of the effectiveness in resisting gastric inflammation. The analysis showed that FF and PR had no significant effect on Y_GIC_, whereas LAR*ZR, CR*ZR, and CR*LAR were the main contributors to increased Y_GIC_, suggesting that ZR, CR, and LAR are likely the key botanical drugs responsible for the anti-inflammatory effect of MHWP. Additionally, ZR*GCR and LAR*GCR had minor enhancing effects on Y_GIC_, indicating that GCR supports the local anti-inflammatory action of MHWP through interaction with the key botanical drugs ZR and LAR (Figure 3a).

ZR demonstrated the most significant reduction in serum Y_IL-1β (S)_ and Y_IL-6 (S)_, while ZR*PR exhibited the strongest decrease in Y_TNF-α (S)_, highlighting the critical role of the botanical drug ZR in alleviating systemic inflammation. Additionally, ZR*FF and ZR*PR contributed to minor reductions in Y_IL-1β (S)_ and Y_IL-6 (S)_, suggesting that FF and PR serve as supporting roles alongside the primary botanical drug in reducing these serum inflammatory markers. Notably, CR had no significant effect on Y_IL-6 (S)_ or Y_TNF-α (S)_, while CR*ZR exhibited a slight reduction in Y_IL-1β (S)_, indicating the botanical drug CR’s auxiliary role in modulating serum IL-1β levels. Similarly, LAR showed no significant effect on Y_IL-1β (S)_ or Y_TNF-α (S)_, but LAR*FF demonstrated a slight reduction in Y_IL-6 (S)_, suggesting LAR’s secondary role in lowering serum IL-6 levels. In contrast, PR^2 and GCR*FF showed slight increases in Y_TNF-α (S)_ (Figure 3b).

Y_SIC_ (Serum Inflammatory Cytokines), a composite index derived from Y_IL-1β (S)_, Y_IL-6 (S)_, and Y_TNF-α (S)_ with weight coefficients of 0.3627, 0.4121, and 0.2252, respectively, serves as a quantitative measure of enhanced holistic anti-inflammatory capacity. LAR, CR, and GCR had no significant effect on Y_SIC_, whereas ZR was the primary contributor to its improvement, underscoring the pivotal role of ZR as a key botanical drug in enhancing systemic anti-inflammatory effects. Additionally, ZR*PR and ZR*FF showed modest positive effects, indicating that PR and FF provide supportive contributions alongside the primary botanical drug. In contrast, PR^2 had a slight negative effect on Y_SIC_ (Figure 3b).

#### 3.1.7 PI3K/Akt/NF-κB protein and mRNA suppression in gastric tissue

MHWP significantly reduced the expression of PI3K, Akt, and NF-κB in ethanol-induced gastric tissues (Supplementary Table S9).

ZR^2 exhibited the strongest suppression of Y_PI3K_, with both ZR^2 and ZR significantly reducing Y_PI3K(mRNA)_, highlighting the central role of the botanical drug ZR in downregulating PI3K. Additionally, GCR^2 and GCR*PR showed minor suppressive effects on Y_PI3K_, while LAR*FF modestly reduced Y_PI3K(mRNA)_, indicating that GCR, PR, LAR, and FF act as auxiliary botanical drugs in PI3K regulation. In contrast, the botanical drug CR caused a slight upregulation of Y_PI3K_ (Figure 3c).

FF had no significant effect on Y_Akt_ or Y_Akt(mRNA)_. However, ZR*PR exhibited a dual regulatory effect, slightly increasing Y_Akt(mRNA)_ but demonstrating the strongest suppression of Y_Akt_. Notably, ZR showed the most pronounced reduction in Y_Akt (mRNA)_, further underscoring its primary role in suppressing Akt expression, with the botanical drug PR acting as a supportive regulator. Additionally, LAR*ZR and ZR*GCR slightly reduced Y_Akt_, while LAR^2 and ZR*CR caused minor decreases in Y_Akt(mRNA)_, suggesting that LAR, GCR, and CR function as secondary botanical drugs (Figure 3d).

ZR exerted the most significant inhibitory effect on both Y_NF-κB (G)_ and its mRNA expression, emphasizing its key role in suppressing NF-κB expression in gastric tissue. Meanwhile, ZR*FF and CR*GCR produced relatively minor reductions in Y_NF-κB (G)_, and ZR*GCR and LAR^2 slightly decreased Y_NF-κB(mRNA) (G)_, indicating that the botanical drugs GCR, CR, FF, and LAR provide supportive roles in gastric NF-κB downregulation. Interestingly, PR^2 caused an upregulation of Y_NF-κB(G)_, while ZR*PR reduced Y_NF-κB(mRNA) (G)_, suggesting that the regulatory effect of PR on gastric NF-κB expression depends closely on its dosage (Figure 3e).

### 3.2 The remediation of liver function favorably contributes to the GMI repair

#### 3.2.1 ALT, AST and TBIL levels in serum

MHWP significantly reduced ethanol-induced elevations in serum levels of AST, ALT, and TBIL (Supplementary Table S10).

**FIGURE 4:**
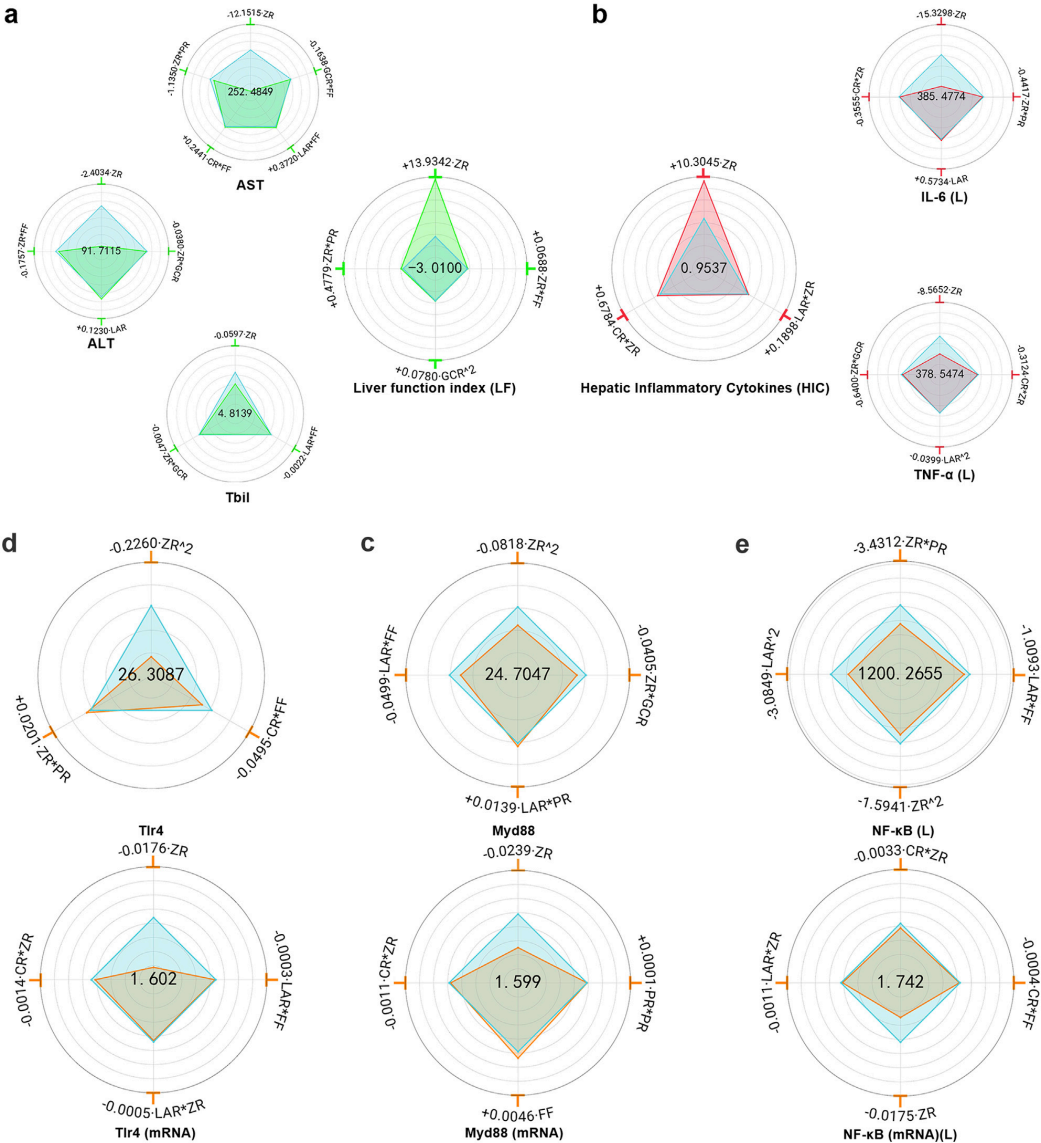
Hierarchical botanical drugs in MHWP regulated hepatic inflammatory factors IL-6(L) and TNF-α(L) via the TLR4/MyD88/NF-κB pathway, modulating liver function. **(a)**, Radar chart for SPRA of Y_ALT_, Y_AST,_ Y_TBIL_ and Y_LF_. **(b)**, Radar chart for SPRA of Y_IL-6(L)_, Y_TNF-α(L)_ and Y_HIC_. **(c)**, Radar chart for SPRA of Y_TLR4_ and Y _TLR4(mRNA)_. **(d)**, Radar chart for SPRA of Y_MyD88_ and Y _MyD88(mRNA)_. **(e)**, Radar chart for SPRA of Y_NF-κB(L)_ and Y _NF-κB(mRNA) (L)_.

ZR exhibited the most significant reduction in Y_AST_, Y_ALT_, and Y_TBIL_, establishing it as the primary botanical drug responsible for MHWP’s hepatoprotective effects. Additionally, ZR*FF and ZR*GCR demonstrated moderate effects in reducing Y_ALT_, indicating that the botanical drugs FF and GCR play supportive roles in its regulation. For Y_AST_, ZR*PR and GCR*FF showed moderate effects, suggesting that PR, GCR, and FF act as auxiliary botanical drugs in its modulation. Regarding Y_TBIL_, ZR*GCR and LAR*FF caused minor reductions, highlighting the botanical drugs GCR, LAR, and FF as complementary roles that enhance the therapeutic efficacy of ZR. Interestingly, CR had no significant effect on Y_ALT_ or Y_TBIL_, while CR*FF caused a slight increase in Y_AST_, indicating that CR is unlikely to contribute meaningfully to liver function regulation (Figure 4a).

Y_LF_ (Liver Function index), a composite index calculated from Y_ALT_, Y_AST_, and Y_TBIL_ with respective weight coefficients of 0.3444, 0.3246, and 0.3310, provided a quantitative assessment of liver function repairing. Regression analysis showed that CR and LAR had no significant effect on Y_LF_, while ZR exhibited the most substantial positive effect, reinforcing its role as the key botanical drug in promoting liver function repair. Additionally, ZR*PR and ZR*FF contributed modestly to Y_LF_, highlighting the supportive roles of PR and FF in hepatoprotection through interaction with the primary botanical drug ZR. Notably, GCR^2 also produced a slight increase in Y_LF_ (Figure 4a).

#### 3.2.2 IL-6 and TNF-α levels in liver tissue

MHWP significantly reduced IL-6 and TNF-α levels in liver tissue (Supplementary Table S11). Regression analysis showed that FF and GCR had no significant effect on Y_IL-6(L)_, while ZR exhibited the most substantial downregulatory effect, establishing it as a key botanical drug in regulating IL-6 levels. Additionally, CR*ZR and ZR*PR moderately reduced Y_IL-6(L)_, indicating that CR and PR played supporting roles in lowering IL-6 levels in the liver. In contrast, LAR displayed a minor increase in Y_IL-6(L)_. For Y_TNF-α (L)_, FF and PR did not significantly affect its levels, whereas ZR demonstrated the strongest suppressive effect, highlighting its pivotal role in reducing TNF-α levels in liver tissue. Moreover, ZR*GCR, LAR^2, and CR*ZR exhibited mild reductions in Y_TNF-α(L)_, suggesting that the botanical drugs GCR, LAR, and CR contributed auxiliary roles in its suppression (Figure 4b).

Y_HIC_ (Hepatic Inflammatory Cytokines), a composite index derived from Y_IL-6(L)_ and Y_TNF-α(L)_ with weight coefficients of 0.5424 and 0.4576, respectively, was used to quantitatively assess the effectiveness in resisting hepatic inflammation. GCR, FF and PR showed no significant effects on Y_HIC_, whereas ZR exhibited the most pronounced positive effect, highlighting its role as the primary botanical drug mediating MHWP’s anti-inflammatory effects on the liver. Additionally, CR*ZR and LAR*ZR exerted secondary positive effects on Y_HIC_, suggesting that the botanical drugs CR and LAR served complementary roles in MHWP’s liver anti-inflammatory mechanisms (Figure 4b).

### 3.3 TLR4/MyD88/NF-κB protein and mRNA suppression in hepatic tissue

MHWP significantly reduced the protein levels and relative mRNA expression of TLR4, MyD88, and NF-κB in liver tissue (Supplementary Table S12).

GCR had no significant effect on Y_TLR4_ or Y_TLR4(mRNA)_. In contrast, ZR^2 showed the most pronounced reduction in Y_TLR4_, while ZR demonstrated the greatest decrease in Y_TLR4(mRNA)_, establishing ZR as a critical botanical drug for lowering hepatic Y_TLR4_ levels. Additionally, CR*FF slightly reduced Y_TLR4_, while CR*ZR, LAR*ZR, and LAR*FF showed minor decreases in Y_TLR4(mRNA)_, suggesting that CR, FF, and LAR serve as auxiliary botanical drugs in reducing TLR4 expression (Figure 4c).

For Y_MyD88_, ZR^2 exhibited the most significant reduction, with ZR producing the greatest decrease in Y_MyD88(mRNA)_, highlighting the pivotal role of botanical drugs ZR in reducing hepatic MyD88 expression. Furthermore, LAR*FF and ZR*GCR showed moderate reductions in Y_MyD88_, while CR*ZR, LAR*ZR, and CR*FF exhibited mild effects on Y_MyD88(mRNA)_. These results suggest that CR, FF, LAR, and GCR act as auxiliary regulators, complementing the primary effects of ZR (Figure 4d).

GCR had no measurable effect on Y_NF-κB (L)_ or its mRNA expression. However, ZR*PR and ZR demonstrated the most pronounced reductions, with ZR showing the strongest decrease in its mRNA expression. These findings underscore the central roles of ZR and PR in modulating NF-κB expression in hepatic tissue. Additionally, LAR^2 and LAR*FF exhibited secondary effects in reducing Y_NF-κB (L)_, while ZR*CR showed a minor effect on its mRNA expression, suggesting that the botanical drugs CR, FF, and LAR play supportive roles in hepatic NF-κB regulation. Conversely, FF and PR^2 slightly increased the mRNA expression of hepatic NF-κB (Figure 4e).

### 3.4 Hepatic function recovery closely linked to MHWP-improved GMI

**FIGURE 5:**
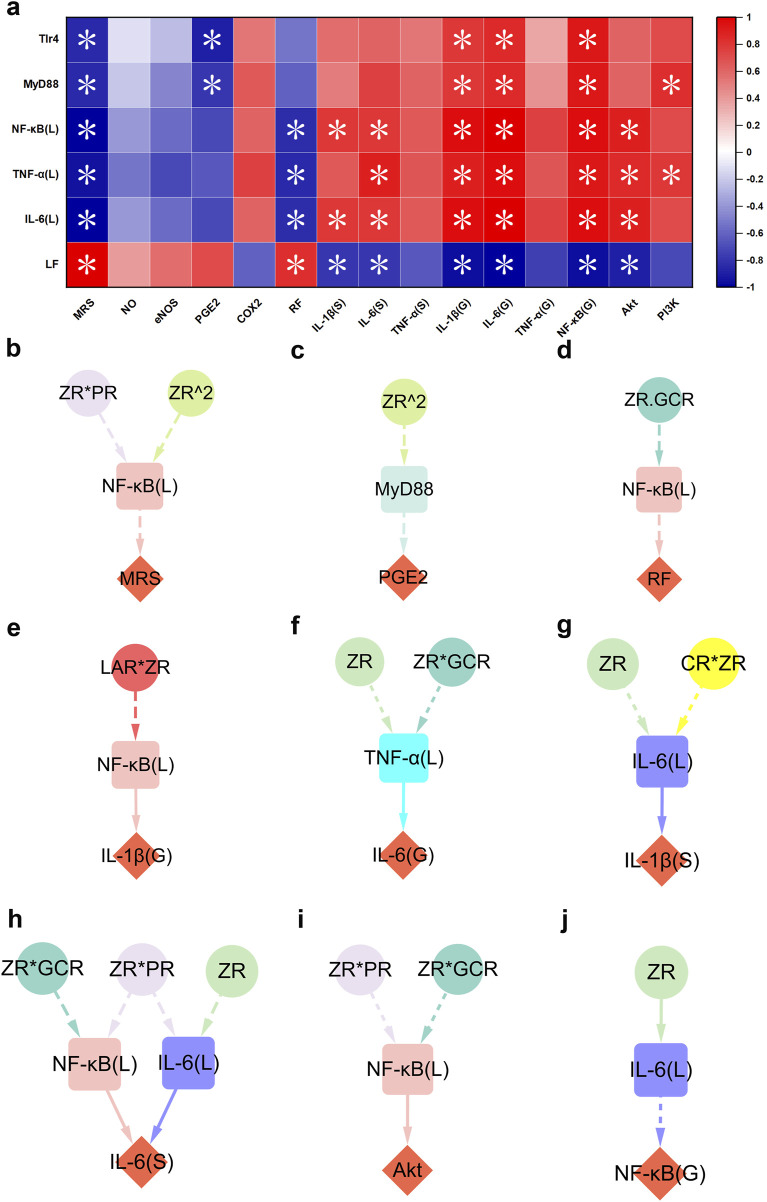
TLR4/MyD88/NF-κB-mediated hepatic function restoration contributed to the process of MHWP-promoted gastric mucosal repair. **(a)**, Heatmap of the correlation between pharmacological niches in TLR4/MyD88/NF-κB-mediated hepatic function restoration and pharmacodynamic niches in gastric mucosal repair. **(b–j)**, Effects of botanical drugs in MHWP on MRS (Fisher.C = 2.720, p = 0.606), PGE2 (Fisher.C = 2.569, p = 0.277), RF (Fisher.C = 1.923, p = 0.750), IL-1β(G) (Fisher.C = 0.356, p = 0.837), IL-6(G) (Fisher.C = 2.482, p = 0.648), IL-1β(S) (Fisher.C = 0.284, p = 0.991), IL-6(S) (Fisher.C = 8.047, p = 0.781), Akt (Fisher.C = 3.605, p = 0.462) and NF-κB(G) (Fisher.C = 0.398, p = 0.820) mediated by hepatic function; circles, denoting botanical drugs, squares, symbolizing liver function, and diamonds, indicating pharmacodynamic niches in gastric mucosal repair.

To investigate the interaction between hepatic and gastric injury, and to elucidate the underlying mechanisms of gastric mucosal repair, we conducted Spearman correlation analyses to assess associations between hepatic and gastric injury biomarkers (Figure 5a). The analysis revealed no significant correlations between the hepatic repair markers examined in this study and NO, eNOS, COX-2, or serum TNF-α (S). These results suggest that these mediators may exert localized effects within the gastric tissue, rather than being directly regulated by hepatic repair processes. Notably, the liver function index (LF) was significantly positively correlated with MRS and RF, and negatively correlated with serum IL-1β (S), serum IL-6 (S), gastric IL-6 (G), NF-κB (G), and Akt, indicating that hepatic function may play a regulatory role in promoting gastric mucosal repair. This reparative effect is likely mediated through suppression of key inflammatory signaling pathways, particularly the reduction in the expression levels of gastric AKT and NF-κB (G). Additionally, hepatic levels of IL-6 (L), TNF-α (L), NF-κB (L), MyD88, and TLR4 were inversely correlated with MRS. In contrast, these markers showed positive correlations with gastric IL-1β (G), IL-6 (G), and NF-κB (G). These findings suggest a potential interplay between hepatic and gastric inflammatory responses, potentially involving modulation of the NF-κB. Of particular interest, hepatic expression levels of IL-6 (L), TNF-α (L), and NF-κB (L) were inversely correlated with RF, while being positively associated with serum IL-6 (S) and gastric Akt expression. Furthermore, hepatic IL-6 (L) and NF-κB (L) were specifically positively correlated with serum IL-6 (S), whereas hepatic TNF-α (L) showed a distinct positive correlation with PI3K expression. Lastly, hepatic expression levels of TLR4 and MyD88 were significantly negatively correlated with gastric PGE2 levels. Notably, hepaticTLR4 also exhibited a positive correlation with gastric PI3K expression. These findings collectively suggest that hepatic inflammation and immune signaling pathways may influence gastric mucosal homeostasis through both systemic and localized regulatory mechanisms.

Building on these findings, a further pSEM analysis was conducted to explore how liver function participates in the MHWP-mediated regulation of GMI. The results highlighted hepatic NF-κB (L), IL-6 (L) TNF-α (L), and MyD88 as key mediators linking botanical interventions to mucosal repair. Specifically, hepatic NF-κB (L), modulated by ZR*PR and ZR^2, contributes to the improvement of MRS (Figure 5b). Furthermore, it is involved in enhancing RF under the influence of ZR*GCR and plays a role in regulating gastric IL-1β (G) in response to LAR*ZR (Figures 5d,e). It is also associated with the regulation of gastric AKT under the combined influence of ZR*PR and ZR*GCR (Figure 5i). Hepatic IL-6 (L), modulated by ZR and CR*ZR, is involved in the suppression of serum IL-1β (S), while also regulating gastric NFκB (G) under the influence of ZR (Figures 5g,j). Notably, hepatic NF-κB (L) and IL-6 (L) jointly regulate serum IL-6 (S), with NF-κB (L) being influenced by ZR*PR and ZR*GCR, and IL-6 (L) by ZR*PR and ZR (Figure 5h). Furthermore, hepatic MyD88, under the influence of ZR^2, contributes to the enhancement of PGE2 levels, while hepatic TNF-α (L), modulated by ZR and ZR*GCR, is involved in the regulation of gastric IL-6 (G) (Figures 5c,f).

### 3.5 Regulation of gut microbiota closely linked to MHWP-improved GMI

MHWP significantly influenced the diversity of gut microbiota in rats with ethanol-induced gastric mucosal injury. Specifically, it reduced the increase in α-diversity caused by ethanol exposure, as demonstrated by metrics such as the Chao1, Shannon, and Simpson indices (Supplementary Figures S1a–c). Furthermore, β-diversity analysis using Bray–Curtis dissimilarity revealed that the gut microbial community in MHWP-treated rats did not separate from the Control but exhibited significant divergence from ethanol-exposed rats (Supplementary Figure S1d). These findings suggest that MHWP may partially restore the dysbiosis of gut microbiota induced by ethanol exposure. This study further analyzed the species composition at the phylum and genus levels to investigate the specific microbial alterations induced by MHWP. The results revealed varying degrees of changes in the relative abundance of key intestinal microorganisms (Supplementary Figures S1e,f). These alterations in microbial composition demonstrate MHWP’s capacity to modulate gut microbiota, supporting the restoration of microbial homeostasis disrupted by ethanol exposure. In addition, the LDA value distribution histogram and the corresponding cladogram, derived from LEfSe (Linear Discriminant Analysis Effect Size), were used to display the microbiota and their taxonomic hierarchies significantly associated with ethanol-induced GMI, as well as those related to MHWP-mediated GMI improvement (Supplementary Figure S2).

**FIGURE 6:**
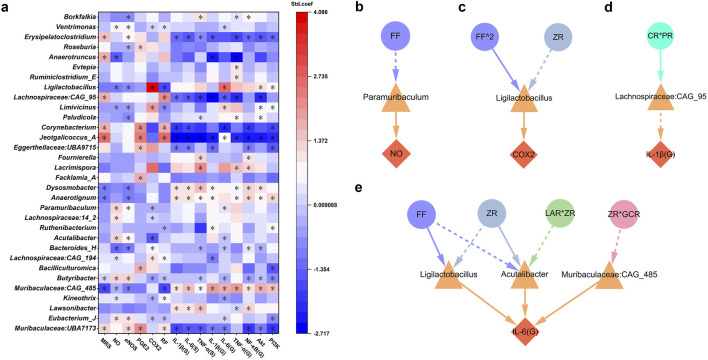
Gut microbiota associated with the botanical drugs in MHWP involved in the process of gastric mucosal repair. **(a)**, PLSR analysis of gut microbiota associated with pharmacodynamic niches in gastric mucosal repair, where * indicates variables with VIP >0.05 in the model. **(b–e)**, Regulatory effects of gut microbiota influenced by specific botanical drugs in MHWP on NO (Fisher.C = 4.812, p = 0.090), COX-2 (Fisher.C = 2.269, p = 0.686), IL-1β(G) (Fisher.C = 3.546, p = 0.170), IL-6(G) (Fisher.C = 28.383, p = 0.340); circles, denoting botanical drugs, triangles, symbolizing liver function, and diamonds, indicating pharmacodynamic niches in gastric mucosal repair.

To identify bacterial genera closely associated with pharmacological niches, this study performed PLSR analysis using discriminative bacterial genera selected by LEfSe, along with various gastric indicators (Figure 6a). The results revealed that genera such as *Jeotgalicoccus_A*, *Corynebacterium*, *Lachnospiraceae:CAG_95*, *Anaerotruncus*, *Erysipelatoclostridium*, and *Muribaculaceae:CAG_485* were significantly associated with improvements in MRS, highlighting their contributory roles in GMI repair. Conversely, *Muribaculaceae:CAG_485*, *Dysosmobacter*, *Anaerotignum*, and *Butyribacter* were linked to reduced MRS, potentially acting as inhibitors of mucosal repair. These findings suggest that these genera, potentially influenced by MHWP, exhibit broad activity across multiple targets within the EGF-NO/PGE2 axis or the PI3K/Akt/NF-κB pathway. Specifically, they modulate the repair process by increasing beneficial genera and decreasing harmful ones. Notably, some genera, despite having no direct effect on MRS, were critical for modulating specific pharmacological niches within the EGF-NO/PGE2 axis and PI3K/Akt/NF-κB pathway. These genera include *Eubacterium_J*, *Kineothrix*, *Lachnospiraceae:CAG_194*, *Bacteroides_H*, *Acutalibacter*, *Lachnospiraceae:14_2*, *Paramuribaculum*, *Limivicinus*, *Ligilactobacillus*, *Roseburia*, and *Ventrimonas*, which influence multiple targets within the EGF-NO/PGE2 axis. Moreover, *Paludicola* specifically influences eNOS; *Bacilliculturomica*, *Facklamia_A*, and *Eggerthellaceae:UBA9715* specifically affect PGE2; and *Ruthenibacterium* specifically affects RF. Additionally, with the exception of *Lachnospiraceae:14_2*, *Facklamia_A*, and *Roseburia*, all of these genera are linked to the anti-inflammatory capacity associated with the regulation of key target expression within the PI3K/AKT/NFκB pathway.

Building on these findings, further analysis was conducted to identify gut microbiota associated with botanical drugs involved in the mucosal repair process. The results demonstrated that *Paramuribaculum*, *Ligilactobacillus*, *Lachnospiraceae:CAG_95*, *Acutalibacter*, and *Muribaculaceae:CAG_485* are key genera mediating the pharmacological effects of botanical interventions. Specifically, *Paramuribaculum* was associated with the regulation of NO production, likely through its linkage to the actions of FF (Figure 6b). *Ligilactobacillus* was found to modulate COX-2 levels in association with FF^2 and ZR (Figure 6c). *Lachnospiraceae:CAG_95* contributed to the regulation of gastric IL-1β (G), likely mediated by the action of CR*PR (Figure 6d). Notably, *Acutalibacter*, *Ligilactobacillus*, and *Muribaculaceae:CAG_485* jointly influenced the regulation of gastric IL-6 (G) (Figure 6e). Among them, *Acutalibacter* was modulated by ZR and ZR*LAR, *Ligilactobacillus* by FF and ZR, and *Muribaculaceae:CAG_485* by ZR*GCR.

### 3.6 Active serum metabolites associated with the herbs in MHWP-improved GMI

**FIGURE 7:**
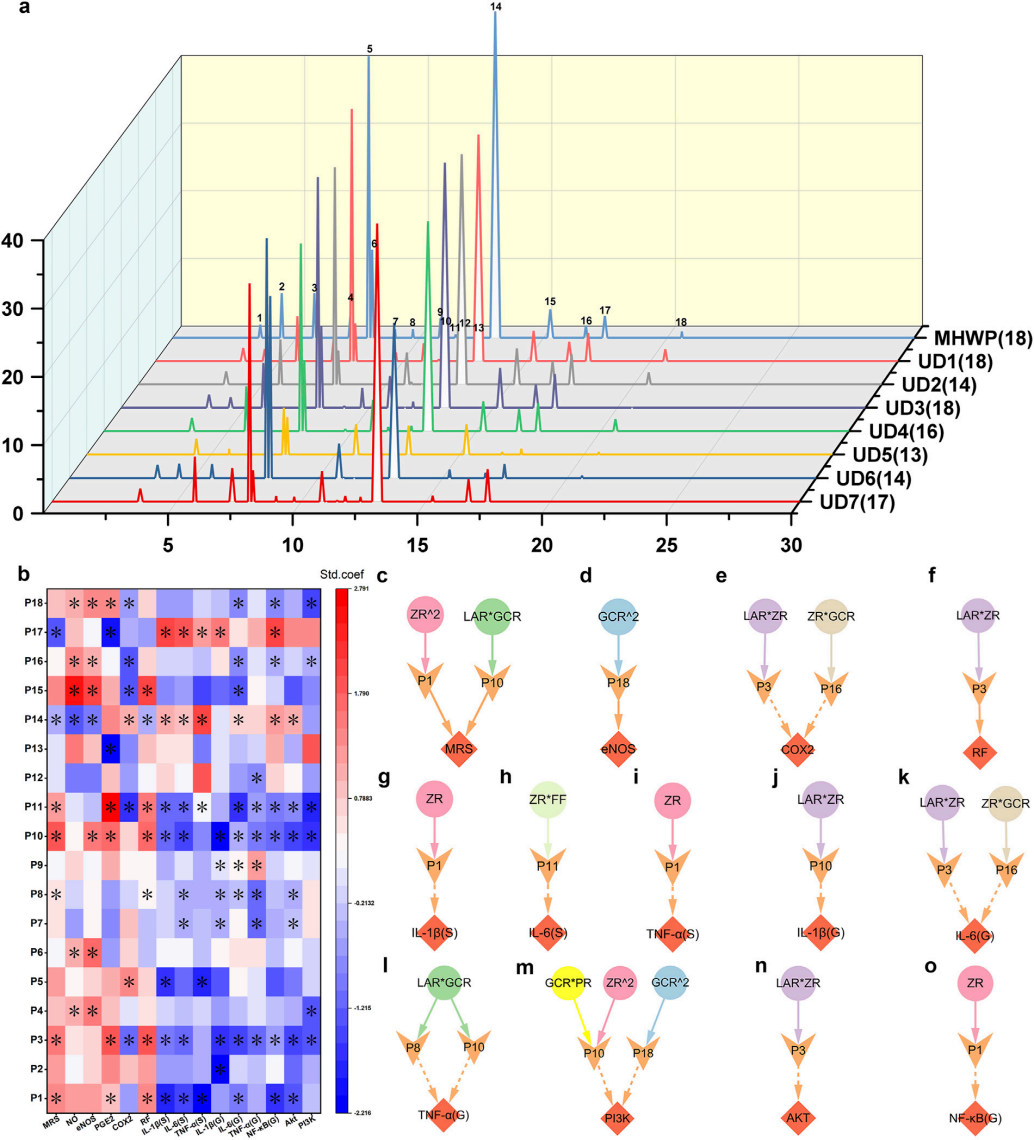
Active serum metabolites associated with the botanical drugs in MHWP involved in the process of gastric mucosal repair. **(a)**, HPLC serum fingerprint analysis of MHWP with various botanical formulations, with the numbers in parentheses indicating the remaining characteristic peaks after subtracting those shared with the control serum. **(b)**, PLSR analysis of characteristic peaks and pharmacodynamic niches, with color indicating standardized coefficients (deeper red for larger positive and deeper blue for larger negative values); * denotes VIP >1. **(c–o)**, Regulatory effects of serum metabolites influenced by specific botanical drugs in MHWP on MRS (Fisher.C = 6.462, p = 0.775), eNOS (Fisher.C = 0.052, p = 0.974), COX-2 (Fisher.C = 13.148, p = 0.215), RF (Fisher.C = 2.855, p = 0.240), IL-1β(S) (Fisher.C = 3.056, p = 0.217), IL-6(S) (Fisher.C = 1.324, p = 0.516), TNF-α(S) (Fisher.C = 0.03, p = 0.985), IL-1β(G) (Fisher.C = 0.244, p = 0.885), IL-6(G) (Fisher.C = 9.492, p = 0.486), TNF-α(G) (Fisher.C = 4.951, p = 0.292), PI3K (Fisher.C = 13.626, p = 0.478), Akt (Fisher.C = 0.876, p = 0.645) and NF-κB(G) (Fisher.C = 2.473, p = 0.290); circles, denoting botanical drugs, V-shaped, symbolizing liver function, and diamonds, indicating pharmacodynamic niches in gastric mucosal repair.

A total of eighteen characteristic peaks were identified in the HPLC fingerprint (Figure 7a, Supplementray Figure S3). To assess their functional significance, PLSR analysis was performed to elucidate their roles in gastric mucosal repair and related signaling pathways (Figure 7b). Among these, P1, P3, P8, P10, P11, P14, and P17 exhibited significant effects on the MRS, influencing multiple pharmacological targets within both the EGF-NO/PGE2 axis and the PI3K/Akt/NF-κB pathway. Notably, P10 and P14 were specifically involved in the regulation of eNOS expression and the enhancement of NO production in gastric tissue. Although peaks P2, P4, P5, P6, P7, P9, P12, P13, P15, P16, and P18 did not directly impact the MRS, they played critical roles in modulating distinct pharmacological niches within the EGF-NO/PGE_2_ axis and PI3K/Akt/NF-κB signaling. Among these, P15, P16, and P18 were particularly notable for influencing multiple targets within the EGF-NO/PGE_2_ axis, highlighting their potential importance in the process of gastric mucosal repair. Additionally, P4 and P6 were specifically associated with the regulation of eNOS expression and NO production; P13 selectively modulated PGE2 levels; and P5 primarily affected COX-2 expression. With the exception of P6 and P13, all of these peaks were linked to anti-inflammatory activity, likely mediated by the modulation of inflammatory cytokine levels, which may be regulated through the expression of key molecular targets within the PI3K/Akt/NF-κB signaling pathway.

To investigate the metabolites corresponding to the 18 serum characteristic peaks in the HPLC fingerprint, this study utilized quadrupole-Orbitrap tandem mass spectrometry combined with a custom database matching approach. The TIC profiles of MHWP-containing serum are presented in Supplementary Figure S4. Each characteristic peak was assigned its corresponding IUPAC name, CAS number, and CID number for metabolites without CAS numbers. The results are provided in the Supplementary Data for ESI-MS file.

Further pSEM analysis identified P1 (CID: 442793, 6-Gingerol), P3 (CID: 5321018, Atractylenolide I), P8 (CID: 11005, Myristic acid), P10 (CID: 14448070, Atractylenolide II), P11 (CID: 117319, 1-Linoleoyl Glycerol), P16 (CID: 168114, 8-Gingerol), and P18 (CID: 637247, Dihydrostilbene base + 3O, 2Prenyl) as key contributors to gastric mucosal repair and inflammatory regulation. These serum metabolites, either originating directly from specific botanical drugs, produced as metabolites of the original components via gut microbiota, or generated as secondary metabolites of microorganisms, exert significant effects on MRS and distinct pharmacological niches. Specifically, P1 and P10 synergistically enhanced the MRS, with P1 being closely associated with the actions of ZR^2 and P10 with the actions of LAR*GCR, underscoring their critical roles in MHWP’s GMI amelioration (Figure 7c). Concurrently, P1 played a pivotal role in regulating serum and gastric inflammatory cytokines, as it reduced serum IL-1β (S), TNF-α (S), and gastric NF-κB (G), in conjunction with the action of ZR (Figures 7g,i,o). Regarding P10, it predominantly regulated inflammatory signaling in gastric tissue. Notably, P10 reduced IL-1β (G) levels in gastric tissue when associated with LAR*ZR (Figure 7j); P10 and P8, both strongly linked to LAR*GCR, decreased TNF-α (G) levels (Figure 7l); and P10 and P18 worked together to downregulate PI3K levels in gastric tissue, with P10 associated with the actions of GCR*PR and ZR^2, and P18 linked to GCR^2 (Figure 7m). Furthermore, P18 upregulated eNOS when associated with the action of GCR^2, suggesting its involvement in vascular homeostasis (Figure 7d). P3 and P16 were shown to downregulate COX-2 and IL-6 (G) levels through their associations with LAR*ZR and ZR*GCR, respectively, with P3 also enhancing RF and reducing Akt levels (Figures 7e,f,k,n). Lastly, P11 reduced IL-6 (S) levels under the influence of ZR*FF (Figure 7h).

## 4 Discussion

GMI presents significant clinical challenges due to its multifactorial etiology and complex pathophysiology, necessitating a comprehensive therapeutic approach (Galura et al., 2019). While conventional treatments primarily alleviate symptoms, they often overlook the critical systemic interactions required for effective and sustained mucosal repair (Yang et al., 2022). In contrast, MHWP employs a multi-targeted strategy by emphasizing *Wei-Qi*, the protective and regulatory energy central to TCM. By addressing the interconnected roles of gastrointestinal, hepatic, and microbiota-mediated pathways, MHWP promotes holistic repair processes. Offering a novel and systemic approach to the management of GMI, MHWP exemplifies the integration of TCM principles with modern biomedical insights, showcasing the strengths of the RELISH framework, which highlights the pivotal role of the gastrointestinal system.

GMI disrupts vascular endothelial cells and microcirculation, leading to mucosal shedding, bleeding, and progression to gastric ulcers (Tarnawski et al., 2012). These pathological processes create multiple therapeutic targets, effectively addressed by MHWP’s formulation, which consists of multiple botanical drugs. MHWP mitigates ethanol-induced GMI by improving microcirculation, enhancing mucosal protection, and promoting regeneration, primarily through enhancement of the EGF-NO/PGE2 axis. Meanwhile, ZR, as the primary botanical drug, demonstrates the strongest effects on the EGF-NO/PGE2 axis, a central pathway in gastric protection and repair. GCR amplifies ZR’s effects, ensuring sustained improvement in microcirculation and epithelial regeneration. Additional botanical drugs of MHWP contribute distinct therapeutic benefits: LAR enhances epithelial regeneration by upregulating EGF and EGFR expression, PR selectively increases PGE2 levels to reinforce the mucosal barrier. In contrast, FF and CR exert regulatory effects on mucosal regeneration and microvascular integrity, respectively, albeit with no direct influence on MRS. In summary, MHWP’s multi-targeted approach synergistically addresses GMI pathophysiology, emphasizing vascular repair, mucosal regeneration, and systemic balance, exemplifying its holistic therapeutic potential.

An atypical finding of this study reveals that COX-2 functions primarily as a pro-inflammatory mediator rather than facilitating PGE2 synthesis (Zhao et al., 2009; Ranjani et al., 2020). Notably, MHWP markedly suppressed the expression of PI3K, AKT, and NF-κB—key signaling molecules in the PI3K/AKT/NF-κB axis, a pathway critically involved in inflammatory regulation. Inhibition of this signaling cascade is strongly correlated with the attenuation of pro-inflammatory cytokine production (El Menyiy et al., 2022; Giammona et al., 2025). Consistent with this, significant reductions in IL-1β, IL-6, and TNF-α levels were observed in both serum and gastric tissues in the present study. This suggests that MHWP likely inhibits inflammation by suppressing the expression of this pathway, thereby promoting optimal tissue repair. Specifically, ZR plays a pivotal role in modulating inflammatory mediators in both gastric tissue and serum by downregulating COX-2 expression and suppressing pro-inflammatory cytokines, including TNF-α, IL-1β, and IL-6. LAR and GCR, through synergistic interactions with other botanical drugs, exert complementary effects on this pathway, enhancing both systemic and localized anti-inflammatory responses. CR, PR, and FF function at distinct stages of the pathway: CR contributes to the regulation of TNF-α(G), IL-1β(S), and Akt; PR supports the modulation of IL-1(S), IL-6(S), and PI3K; and FF provides auxiliary regulation of PI3K, NF-κB, IL-1(S), and IL-6(S). This precise hierarchical structure of MHWP ensures a coordinated multi-targeted approach, effectively modulating inflammatory pathways at systemic and localized levels to restore balance and promote gastric mucosal repair.

It is important to note that certain botanical drugs exhibited slight negative effects—either individually, as quadratic terms, or through interaction terms—on specific regulatory niches within the EGF–NO/PGE2 axis and PI3K/AKT/NFκB pathway in this study. However, these effects were not predominant and did not undermine the overall therapeutic efficacy of the formula. On the contrary, they contributed positively to the improvement of MRS, highlighting the inherent complexity and multi-dimensionality of TCM formulas. These minor negative effects may be better understood within the theoretical framework of TCM, which emphasizes systemic balance rather than the linear maximization of individual biological pathways. In this context, the negative effects observed with certain botanical drugs may reflect intrinsic regulatory mechanisms within the formula that serve to prevent overstimulation of specific biological pathways. A key example is the significant downregulation of the eNOS/NO and the moderate upregulation of COX-2 expression by the effect of FF, which may be associated with its potential role in mitigating oxidative stress caused by excessive NO levels and preventing the over-suppression of inflammatory responses (Sharma et al., 2007; Kalinski, 2012; Talib et al., 2020).

To summary briefly, MHWP adopts a multi-targeted therapeutic approach to address the complex pathophysiology of GMI, focusing on vascular repair, mucosal regeneration, and the modulation of systemic inflammation. This approach highlights MHWP’s potential to bridge traditional and biomedical paradigms by aligning with TCM’s RELISH framework and integrating holistic principles with modern molecular pathways to promote systemic balance and healing.

Beyond its localized effects, MHWP demonstrates systemic benefits, particularly in protecting liver function under ethanol-induced stress. The liver plays a crucial role in maintaining systemic homeostasis and mediating the body’s response to gastric injury, particularly under conditions of ethanol-induced stress (Arteel, 2012). As the primary site for ethanol metabolism, it is integral to systemic inflammatory regulation and tissue repair (Hyun et al., 2021). In this study, MHWP was found to suppress hepatic expression of TLR4, MyD88, and NF-κB—key signaling molecules that have been previously identified as central mediators of liver inflammatory responses (Chen et al., 2021). This suppression was further consistent with the observed downregulation of pro-inflammatory cytokines IL-6 and TNF-α, which likely contributed to the amelioration of hepatocellular injury, as indicated by reduced serum levels of ALT, AST, and TBIL. ZR emerges as the pivotal botanical drug, primarily regulating liver function markers and supporting the suppression of inflammatory cytokines levels. Complementing ZR’s effects, FF and GCR assist in modulating these markers, enhancing their therapeutic impact. LAR plays an additional role by reducing TBIL levels and promoting systemic cytokine reduction, which contributes to the maintenance of systemic balance. Furthermore, CR and FF augment ZR’s suppression of inflammatory indicators along the TLR4/MyD88/NF-κB pathway, while PR and GCR specifically target hepatic IL-6 (L), NF-κB (L), and MyD88, creating a synergistic network of actions that contribute to both mucosal repair and systemic homeostasis.

In this study, the interplay between hepatic reactions and gastric inflammatory pathways, mediated by systemic and gastric cytokines, underpins MHWP’s therapeutic effects, highlighting the coordinated regulation of liver and stomach inflammation for effective gastrointestinal repair and systemic balance. This coordinated regulation has also been increasingly recognized in existing research as vital for achieving gastrointestinal repair and systemic homeostasis (Miyajima et al., 2001; Albillos et al., 2020). More specifically, the expression of the hepatic TLR4/MyD88/NF-κB pathway and hepatic inflammatory cytokines plays a central role in orchestrating systemic inflammatory responses, which in turn may promote gastric mucosal repair. Key hepatic cytokines—particularly NF-κB(L) and IL-6(L)—are essential for linking liver-derived signals to the enhancement of gastric mucosal integrity. Notably, NF-κB(L), primarily modulated by ZR and PR, contributes to improvements in MRS, suppresses gastric IL-1β(G), and inhibits Akt expression. In parallel, hepatic IL-6(L), largely influenced by ZR, reduces gastric NF-κB(G) expression and suppresses systemic cytokines such as serum IL-1β(S). Moreover, the synergistic action of hepatic NF-κB(L) and IL-6(L) mediates the inhibitory effects of ZR, PR and GCR on serum IL-6(S). Furthermore, hepatic TNF-α(L), modulated by ZR and GCR, contributes to the suppression of gastric IL-6(G). These findings highlight MHWP’s ability to integrate liver-derived and gastric inflammatory pathways, supporting the maintenance of systemic homeostasis and facilitating integrated gastrointestinal repair. It is worth noting that this study primarily focuses on data integration and statistical analysis to investigate the mediating role of liver function in the relationship between botanical drug and gastric mucosal repair under MHWP intervention. This approach provides valuable insights; however, more in-depth and refined experimental designs are needed to further elucidate the underlying mechanisms and to validate the findings in a more controlled and systematic manner.

Beyond protecting liver function under ethanol-induced gastric mucosal stress, gut microbiota in this study plays a critical role in MHWP’s systemic therapeutic benefits. Recent research has demonstrated that gut microbiota play a crucial role in promoting gastric mucosal repair through dynamic interactions with the host’s immune system, metabolic pathways, and mucosal integrity (Ding et al., 2024). In the present study, *Ligilactobacillus*, associated with the actions of FF and ZR, was found to positively regulate COX-2 expression. This aligns with previous research showing that *Lactobacillus* species, can induce COX-2 and PGE2 production, thereby promoting mucosal repair and preventing excessive suppression of inflammatory responses (Uribe et al., 2018). Additionally, *Ligilactobacillus* was found to cooperatively upregulate gastric IL-6(G) with Acutalibacter and *Muribaculaceae:CAG 485*, with Acutalibacter associated with ZR and LAR, and *Muribaculaceae:CAG 485* linked to ZR and GCR. This finding is consistent with earlier studies showing that *Ligilactobacillus* can activate IL-6 synthesis in Caco-2 epithelial cells via the NF-κB and p38 MAPK signaling pathways (Jiang et al., 2012). Moreover, the activity of *Acutalibacter* has been associated with gastrointestinal inflammation (Gong et al., 2025). Although research on *Muribaculaceae:CAG 485* is still limited, it has been reported to respond to ginger, and to participate in host metabolic and immune regulation (Guo et al., 2021). Furthermore, *Lachnospiraceae:CAG 95*, which showed a positive association with CR and PR, was involved in the downregulation of IL-1β. Previous studies have shown that members of the *Lachnospiraceae* family can reduce IL-1β expression by producing short-chain fatty acids (SCFAs), which activate G-protein-coupled receptors (GPRs) and inhibit NF-κB signaling (Liu et al., 2021). Finally, *Paramuribaculum*, associated with FF, was found to potentially contribute to NO production. It is known to degrade complex polysaccharides and synthesize SCFAs, which in turn regulate mucosal immune homeostasis and NO biosynthesis, likely through the inducible nitric oxide synthase (iNOS) pathway (Parada Venegas et al., 2019; Liu et al., 2021; Zhu et al., 2024; Shimizu et al., 2025). Therefore, the reduction in NO levels observed in association with FF may result from suppressed *Paramuribaculum* metabolic activity and diminished SCFA-mediated NO signaling.

These gastric mucosal repair effects of MHWP, based on the synergistic interactions among hepatic inflammatory responses and gut microbiota, exhibit a complex interplay with the serum active metabolites. Previous research has demonstrated that the dynamics of serum metabolites induced by TCM interventions serve as a foundational component for facilitating repair through systemic regulation (Zhou et al., 2024). In this study, eighteen serum characteristic peaks were identified in the HPLC fingerprint of MHWP, highlighting their roles in gastric mucosal repair via targeted pharmacological pathways. Further PLSR and pSEM analysis revealed that P1, P3, P8, P10, P11, P16, and P18, through their association with the actions of specific botanical drugs in MHWP, target the EGF-NO/PGE2 and PI3K/Akt/NF-κB pathways, contributing to the improvement of GMI. Among these, P1 (6-Gingerol) primarily influenced by ZR, and P10 (Atractylenolide II) closely associated with LAR, ZR, and GCR, were pivotal in regulating systemic inflammation and mucosal repair. Furthermore, P3 (Atractylenolide I) and P16 (8-Gingerol), linked to LAR, ZR, and GCR, modulated COX-2, RF and IL-6(G), underscoring their supportive roles in mitigating local gastric inflammation. These four serum metabolites were reported to contribute to anti-inflammatory effects and mucosal repair in previous studies (Xie et al., 2023; Pazmandi et al., 2024), further highlighting the essential roles of the botanical drugs ZR and LAR. Moreover, P18 (Dihydrostilbene base + 3O, 2Prenyl), associated with GCR, was found to regulate eNOS and PI3K pathways. This dihydrostilbene derivative has shown potential anti-hepatic fibrosis properties by inhibiting hepatic stellate cell proliferation, as reported in studies on *Glycyrrhiza uralensis* leaves (Fan et al., 2019). However, similar metabolites from *Glycyrrhiza uralensis* roots remain underexplored. These findings underscore the multi-component, multi-target characteristics of MHWP, emphasizing its holistic and systemic therapeutic potential driven by the coordinated actions of key botanical drugs such as ZR, LAR, GCR, and FF in regulating inflammatory pathways and promoting gastric mucosal repair.

Therefore, MHWP’s therapeutic effects are mediated through its ability to modulate hepatic inflammatory responses, interact with gut microbiota, and influence targeted serum metabolites, creating a comprehensive network of pathways. By improving microcirculation, regulating systemic inflammation, and promoting epithelial regeneration, MHWP effectively utilizes the EGF-NO/PGE2 axis to accelerate mucosal repair and modulates the expression of the PI3K/Akt/NF-κB pathway to regulate inflammatory cytokines in addressing GMI. These mechanisms not only facilitate localized mucosal repair but also contribute to systemic homeostasis. Building on this multi-targeted therapeutic strategy, the RELISH framework offers a structured approach that integrates TCM principles with modern biomedical insights. RELISH emphasizes the holistic nature of MHWP’s therapeutic approach by underscoring *Wei-Qi* reinforcement, a central TCM concept that highlights the interconnected roles of gastrointestinal health, immune regulation, and tissue repair. MHWP’s ability to integrate liver-derived and gastric inflammatory pathways aligns with the TCM concept of *Gan-Pi* Regulation, which highlights the dynamic relationship between the liver and the gastrointestinal digestive system’s joint regulation, beneficial for maintaining internal balance. Moreover, MHWP’s influence on gut microbiota supports *Pi*’s function in digestion and nutrient absorption, reinforcing *Wei-Qi*. Furthermore, serum active metabolites—either directly derived from botanical drugs or indirectly produced via liver and gut microbiota metabolism—contribute significantly to this process. Transported via the bloodstream, these metabolites act on specific targets to enhance gastric mucosal repair through systemic regulation, further aligning with the *Gan-Pi Regulation*. Overall, RELISH advances a multi-targeted and synergistic therapeutic model by integrating traditional concepts into a biomedical framework. This structured approach not only addresses localized repair but also promotes systemic homeostasis, showcasing how MHWP exemplifies the RELISH framework’s potential to achieve sustainable and comprehensive healing.

In conclusion, MHWP’s therapeutic efficacy arises from its multi-targeted strategy, which integrates hepatic inflammatory modulation, gut microbiota interactions, and serum metabolite dynamics into a comprehensive network of pathways. By leveraging the EGF-NO/PGE2 and PI3K/Akt/NF-κB pathways, MHWP improves microcirculation, regulates systemic inflammation, and promotes epithelial regeneration, addressing both localized gastric mucosal repair and systemic homeostasis. These findings align with the RELISH framework, integrating TCM principles like *Wei-Qi* reinforcement and *Gan-Pi Regulation* with modern biomedical insights, highlighting the interconnected roles of gastrointestinal health, immune regulation, and tissue repair in a synergistic therapeutic model.

Although this study provides valuable insights into the potential therapeutic effects of MHWP on gastric mucosal injury, several limitations must be acknowledged. Firstly, this study only measured the protein and mRNA expression levels, and the activation of the PI3K/Akt/NF-κB pathway and TLR4/MyD88/NFκB pathway was not directly validated through functional assays such as Western blotting or phosphorylation assays, which are essential for confirming pathway activation and downstream effects. Future studies should include these assays to assess phosphorylated proteins like p65, p-Akt, and p-PI3K, providing more conclusive evidence of pathway involvement. Furthermore, more in-depth mechanistic studies are needed to fully understand how MHWP influences cellular processes, such as apoptosis, cell migration, and proliferation, in the context of gastric mucosal injury. Additionally, long-term efficacy and safety studies are also essential, as this study only assessed short-term effects. Chronic conditions like gastric mucosal injury require long-term data to evaluate the sustainability of MHWP’s therapeutic benefits and potential side effects. Addressing these limitations in future research will provide a more holistic view of MHWP’s mechanisms and its clinical application.

## Data Availability

The data presented in the study are deposited in the NCBI (SRA) repository, accession number PRJNA1283379, accession link https://www.ncbi.nlm.nih.gov/sra/?term=PRJNA1283379.

## Supplementary material

Supplementary Table S1
Supplementary Data for ESI-MS file.

Supplementary Table S2
Source Data of Figures.

Supplementary Table S3
Source Data of Supplementary Figure S1.

Supplementary Table S4
Source Data of Supplementary Figure S2.

Supplementary Table S5
Entropy Weight Calculation Data.

Supplementary Presentation S1
R code for LASSO Regression and SPRA.

Supplementary Presentation S2
R code for PLSR analysis.

Supplementary Presentation S3
R code for pSEM.

Supplementary Presentation S4
Supplementary information, including Supplementary method, Supplementary Tables S1-S12, and Supplementary Figures S1-S4.
