# Supplementary material for: Gastric mucosal repair by Men’s Huwei Powder via EGF-NO/PGE2-PI3K-TLR4 in RELISH: Restoring Equilibrium through long-term integration of synergistic health
Source: Front Pharmacol. 2025 Jul 11;16:1594089. doi: 10.3389/fphar.2025.1594089 (PMC12289676; doi:10.3389/fphar.2025.1594089)
Supplement: Supplementary file 3 [file Presentation1.pdf]

The r code for LASSO Regression and SPRA (Example: EGF)

1. Install Required Packages

```
install.packages("readxl")
install.packages("car")
install.packages("glmnet")

library(readxl)
library(car)
library(glmnet)
```

2. Data Import and Numeric Conversion

```
file_path <- "C:/Users/73668/Desktop/Dataset used in LASSO&SPRA.xlsx"

X <- read_excel(file_path)
X <- as.data.frame(lapply(X, function(col) {
   if (is.character(col) || is.factor(col)) {
      as.numeric(as.character(col))    # Convert character or factor variables to numeric
   } else {
      col    # Retain numeric columns
   }
}))
```

3. Define Independent Variables and Standardize Data

```
x <- as.data.frame(scale(X[, 1:27]))    # Standardize independent variables
y <- as.data.frame(scale(X[, 28:68])) # Standardize dependent variables
```

4. LASSO Regression

```
# Set lambda = 0.01
lambda <- 0.01

# Perform LASSO regression
la.eq <- glmnet(x, y$EGF, lambda = lambda,
                         family = 'gaussian',
                         intercept = FALSE, alpha = 1)

# If alpha = 0, Ridge regression is performed; setting alpha between 0 and 1 implements
Elastic Net.

# Display coefficient estimates (for lambda = 0.01)
la.eq$beta[,1]

# Visualize the variable selection process dynamically
```

```
la.eq <- glmnet(x, y$EGF, family = "gaussian",
                intercept = FALSE, alpha = 1)

# Plot LASSO regression paths
plot(la.eq, xvar = "lambda", label = FALSE)

# Perform cross-validation and select optimal lambda
mod_cv <- cv.glmnet(x = x, y = y$EGF, family = "gaussian",
                    intercept = FALSE, alpha = 1)    # Default nfolds = 10

# Plot cross-validation results
plot(mod_cv)

# Identify optimal lambda values
print(paste("Lambda.min:", mod_cv$lambda.min, "Log(lambda.min):",
log(mod_cv$lambda.min)))
print(paste("Lambda.1se:", mod_cv$lambda.1se, "Log(lambda.1se):",
log(mod_cv$lambda.1se)))

# Select lambda.min as the optimal lambda
best_lambda <- mod_cv$lambda.min
best_lambda

# Estimate coefficients for the final model
best_model <- glmnet(x, y$EGF, alpha = 1, lambda = best_lambda)
coef(best_model)

# Predict using the best model
mat <- as.matrix(data.frame(x))
y_predicted <- predict(best_model, s = best_lambda, newx = mat)

# Compute SST (total sum of squares) and SSE (sum of squared errors)
sst <- sum((y$EGF - mean(y$EGF))^2)
sse <- sum((y_predicted - y$EGF)^2)

# Calculate R-squared
rsq <- 1 - sse / sst
rsq
```

5. Identify Significant Variables via Stepwise Regression

```
# Define an empty model with only an intercept
empty_model <- lm(EGF ~ 1, data = X)

# Perform stepwise regression to identify significant variables
```

```
stepwise_model <- step(empty_model,
                       scope = list(upper = ~ . + CR.CR + CR.ZR + CR.FF +
LAR.FF + ZR.GCR),
                       data = X,
                       direction = "both")
```

6. Model Fitting

```
# Fit the final selected model
selected_model_EGF <- lm(EGF ~ LAR.FF + CR.ZR + ZR.GCR + CR.CR, data = X)

# Display model summary
summary(selected_model_EGF)
```
