# Supplementary material for: Gastric mucosal repair by Men’s Huwei Powder via EGF-NO/PGE2-PI3K-TLR4 in RELISH: Restoring Equilibrium through long-term integration of synergistic health
Source: Front Pharmacol. 2025 Jul 11;16:1594089. doi: 10.3389/fphar.2025.1594089 (PMC12289676; doi:10.3389/fphar.2025.1594089)
Supplement: Supplementary file 4 [file Presentation4.pdf]

Supplementary Information

Supplementary method

DNA extraction

Total genomic DNA samples were extracted using the OMEGA Soil DNA Kit (M5635-02) (Omega Bio-Tek, Norcross, GA, USA), following the manufacturer's instructions, and stored at -20 °C prior to further analysis. The quantity and quality of extracted DNAs were measured using a NanoDrop NC2000 spectrophotometer (Thermo Fisher Scientific, Waltham, MA, USA) and agarose gel electrophoresis, respectively.

16S rRNA gene amplicon sequencing

PCR amplification of the bacterial 16S rRNA genes V3–V4 region was performed using the forward primer 338F (5'-ACTCCTACGGGAGGCAGCA-3') and the reverse primer 806R (5'-GGACTACHVGGGTWTCTAAT-3'). Sample-specific 7-bp barcodes were incorporated into the primers for multiplex sequencing. The PCR components contained 5 μl of buffer (5×), 0.25 μl of Fast pfu DNA Polymerase (5U/μl), 2 μl (2.5 mM) of dNTPs, 1 μl (10 uM) of each Forward and Reverse primer, 1 μl of DNA Template, and 14.75 μl of ddH2O. Thermal cycling consisted of initial denaturation at 98 °C for 5 min, followed by 25 cycles consisting of denaturation at 98 °C for 30 s, annealing at 53 °C for 30 s, and extension at 72 °C for 45 s, with a final extension of 5 min at 72 °C. PCR amplicons were purified with Vazyme VAHTSTM DNA Clean Beads (Vazyme, Nanjing, China) and quantified using the Quant-iT PicoGreen dsDNA Assay Kit (Invitrogen, Carlsbad, CA, USA). After the individual quantification step,

amplicons were pooled in equal amounts, and pair-end 2*250 bp sequencing was performed using the Illlumina Nova Seq platform with NovaSeq 6000 SP Reagent Kit (500 cycles).

Sequence analysis

Microbiome bioinformatics were performed with QIIME2 2022.11(Bolyen et al., 2018) with slight modification according to the official tutorials. Briefly, raw sequence data were demultiplexed using the demux plugin following by primers cutting with cut adapt plugin(Martin and Stocchi, 2011). Sequences were then quality filtered, denoised, merged and chimera removed using the DADA2 plugin(Callahan et al., 2016). Non-singleton amplicon sequence variants (ASVs) were aligned with MAFFT(Katoh et al., 2002) and used to construct a phylogeny with FastTree2(Price et al., 2010). Alpha-diversity metrics (Chao1(Chao, 1984), Shannon(Shannon, 1948), Simpson(Simpson, 1949), beta diversity metrics (Bray-Curtis dissimilarity) were estimated using the diversity plugin. Taxonomy was assigned to ASVs using the classify-sklearn naïve Bayes taxonomy classifier in feature-classifier plugin(Bokulich et al., 2018)against the SILVA Release 138 Database(Kõljalg et al., 2013). LEfSe (Linear discriminant analysis effect size) was performed to detect differentially abundant taxa across groups using the default parameters(Segata et al., 2011).

Reference

Bokulich, N.A., Kaehler, B.D., Rideout, J.R., Dillon, M., Bolyen, E., Knight, R., et al. (2018). Optimizing taxonomic classification of marker-gene amplicon sequences with QIIME 2's q2-feature-classifier plugin. *Microbiome* 6(1), 90. doi: 10.1186/s40168-018-0470-z.

Bolyen, E., Rideout, J.R., Chase, J., Pitman, T.A., Shiffer, A., Mercurio, W., et al. (2018). An Introduction to Applied Bioinformatics: a free, open, and interactive text. *J Open Source Educ* 1(5). doi: 10.21105/jose.00027.

Callahan, B.J., Sankaran, K., Fukuyama, J.A., McMurdie, P.J., and Holmes, S.P. (2016). Bioconductor Workflow for Microbiome Data Analysis: from raw reads to community analyses. *F1000Res* 5, 1492. doi: 10.12688/f1000research.8986.2.

Chao, A. (1984). Non-parametric estimation of the classes in a population. *Scandinavian Journal of Statistics* 11, 265-270. doi: 10.2307/4615964.

Katoh, K., Misawa, K., Kuma, K., and Miyata, T. (2002). MAFFT: a novel method for rapid multiple sequence alignment based on fast Fourier transform. *Nucleic Acids Res* 30(14), 3059-3066. doi: 10.1093/nar/gkf436.

Kõljalg, U., Nilsson, R.H., Abarenkov, K., Tedersoo, L., Taylor, A.F., Bahram, M., et al. (2013). Towards a unified paradigm for sequence-based identification of fungi. *Mol Ecol* 22(21), 5271-5277. doi: 10.1111/mec.12481.

Martin, S.T., and Stocchi, L. (2011). Laparoscopic colorectal resection in the obese patient. *Clin Colon Rectal Surg* 24(4), 263-273. doi: 10.1055/s-0031-1295690.

Price, M.N., Dehal, P.S., and Arkin, A.P. (2010). FastTree 2--approximately maximum-likelihood trees for large alignments. *PLoS One* 5(3), e9490. doi: 10.1371/journal.pone.0009490.

Segata, N., Izard, J., Waldron, L., Gevers, D., Miropolsky, L., Garrett, W.S., et al. (2011). Metagenomic biomarker discovery and explanation. *Genome Biology* 12(6), R60. doi: 10.1186/gb-2011-12-6-r60.

Shannon, C.E. (1948). A Mathematical Theory of Communication. 27(3), 379-423. doi: https://doi.org/10.1002/j.1538-7305.1948.tb01338.x.

Simpson, E.H. (1949). Measurement of Diversity. *Nature* 163(4148), 688-688. doi: 10.1038/163688a0.

Supplementary Table

Supplementary Table S1 Botanical drugs in Men's Huwei Powder (MHWP)

Chinese name	Plant (Latin name)	Scientific name
**干姜 (Ganjiang)**	*Zingiber officinale* Rosc.	Zingiberis Rhizoma (ZR)
**甘草 (Gancao)**	*Glycyrrhiza uralensis* Fisch.	Glycyrrhizae Radix et Rhizoma (GCR)
**党参 (Dangshen)**	*Codonopsis pilosula* (Franch.) Nannf.	Codonopsis Radix (CR)
**连翘 (Lianqiao)**	*Forsythia suspensa* (Thunb.) Vahl.	Forsythia Fruit (FF)
**白术 (Baibiandou)**	*Atractylodes macrocephala* Koidz.	Largehead Atractylodes Rhizome (LAR)
**姜半夏 (Jiangbanxia)**	*Pinellia ternate* (Thunb.) Ten. ex Breitenb.	Pinelliae Rhizoma Praeparatum Cum Zingibere Et Alumine (PR)

Supplementary Table S2 Botanical drug's grammage in Uniform Design formulas (UD1-UD7) and original MHWP.

	ZR	GCR	LAR	CR	FF	PR
**MHWP**	4	6	9	6	4	6
**UD1**	5	10	15	10	5	10
**UD2**	3	12	0	8	1	4
**UD3**	2	2	18	6	0	8
**UD4**	6	6	12	0	2	2
**UD5**	0	8	6	2	3	12
**UD6**	4	0	3	4	6	6
**UD7**	1	4	9	12	4	0

Supplementary Table S3 MHWP significantly improved gastric mucosal injury.

Group	UI	HS
**Control**	0.00 ± 0.00^{c}	0.00 ± 0.00^{c}
**Model**	26.20 ± 2.59^{a}	12.20 ± 1.79^{a}
**MHWP**	9.40 ± 1.14^{b}	5.60 ± 1.14^{b}

Note: Values represent the mean±SD (n = 5). Groups were compared for statistical significance, with different superscript letters indicating significant differences between groups ($p < 0.05$). The superscript letters in Supplementary Tables 3 to 12 denote significant differences between groups, with each letter representing a distinct group comparison. Control represents the baseline group, Model is the ethanol exposure group, and MHWP

represents the MHWP treatment group. This notation applies throughout the manuscript.

Supplementary Table S4 MHWP significantly enhanced eNOS expression and serum NO production.

Group	NO	eNOS	eNOS(mRNA)
Control	338.76±13.27[a]	69.69±0.37[b]	1.00±0.12[a]
Model	176.80±8.94[c]	58.08±1.15[c]	0.26±0.06[c]
MHWP	302.81±2.79[b]	77.54±1.46[a]	0.51±0.12[b]

Supplementary Table S5 MHWP significantly increased gastric PGE2 levels.

Group	PGE2
Control	457.84±26.06[a]
Model	257.72±22.94[c]
MHWP	377.85±7.66[b]

Supplementary Table S6 MHWP significantly enhanced EGF and EGFR expression levels.

Group	EGF	EGF(mRNA)	EGFR	EGFR(mRNA)
Control	438.57±22.23[a]	1.00±0.07[a]	33.52±0.85[a]	1.00±0.05[a]
Model	266.11±20.45[c]	0.32±0.04[c]	21.27±1.39[c]	0.32±0.05[c]
MHWP	352.84±18.11[b]	0.46±0.09[b]	27.28±3.04[b]	0.54±0.10[b]

Supplementary Table S7 MHWP significantly reduced gastric COX-2 overexpression.

Group	COX2	COX2(mRNA)
Control	55.29±6.94[b]	1.00±0.11[c]
Model	143.07±6.31[a]	2.97±0.11[a]
MHWP	60.29±7.68[b]	2.22±0.14[b]

Supplementary Table S8 MHWP significantly reduced IL1β, IL6, and TNFα levels in gastric tissue and serum.

Group	IL-1β(G)	IL-6(G)	TNF-α(G)	IL-1β(S)	IL-6(S)	TNF-α(S)
Control	124.58±16.86[c]	12.29±0.96[c]	101.65±6.71[c]	330.09±7.04[b]	30.72±0.61[c]	177.43±7.91[c]
Model	622.66±47.81[a]	62.87±3.96[a]	384.08±18.92[a]	556.85±12.61[a]	51.66±0.77[a]	403.60±5.71[a]
MHWP	466.36±21.26[b]	44.51±2.59[b]	296.77±11.82[b]	333.46±2.45[b]	33.79±0.91[b]	202.33±5.43[b]

Supplementary Table S9 MHWP significantly downregulated the expression of the PI3K, AKT and NF-κB in gastric tissue.

Group	PI3K	PI3K(mRNA)	Akt	Akt(mRNA)	NF-κB(G)	NF-κB(mRNA)(G)
Control	11.57 ± 2.66[c]	1.00±0.08[c]	10.49 ± 2.35[c]	1.00±0.03[c]	268.61 ± 36.69[c]	1.01±0.07[c]
Model	64.85 ± 2.79[a]	2.72±0.16[a]	35.77 ± 2.26[a]	1.94±0.22[a]	1964.96 ± 59.47[a]	2.59±0.12[a]
MHWP	28.41 ± 3.11[b]	2.10±0.11[b]	26.42 ± 0.53[b]	1.43±0.15[b]	1260.91 ± 72.91[b]	1.64±0.07[b]

Supplementary Table S10 MHWP significantly improved liver function.

Group	ALT	AST	TBIL
Control	73.42±1.56[c]	233.54±9.11[b]	3.38±0.37[c]
Model	96.44±1.74[a]	302.30±3.98[a]	5.36±0.21[a]
MHWP	82.28±1.62[b]	227.17±4.64[b]	4.44±0.38[b]

Supplementary Table S11 MHWP significantly reduces the expression of hepatic inflammatory cytokines.

Group	IL-6(L)	TNF-α(L)
Control	34.96±1.50[b]	240.14±9.76[b]
Model	47.81±0.66[a]	421.23±4.41[a]
MHWP	28.65±1.89[c]	229.08±6.90[b]

Supplementary Table S12 MHWP significantly downregulated the expression of the TLR4, MyD88 and NF-κB in hepatic tissue.

Group	TLR4	TLR4(mRNA)	MyD88	MyD88(mRNA)	NF-κB(L)	NF-κB(mRNA) (L)
Control	11.57 ± 2.66[c]	1.00±0.08[c]	10.49 ± 2.35c	1.00±0.03[c]	268.61 ± 36.69[c]	1.01±0.07[c]
Model	64.85 ± 2.79[a]	2.72±0.16[a]	35.77 ± 2.26[a]	1.94±0.22[a]	1964.96 ± 59.47[a]	2.59±0.12[a]
MHWP	28.41 ± 3.11[b]	2.10±0.11[b]	26.42 ± 0.53[b]	1.43±0.15[b]	1260.91 ± 72.91[b]	1.64±0.07[b]

Supplementary Figure

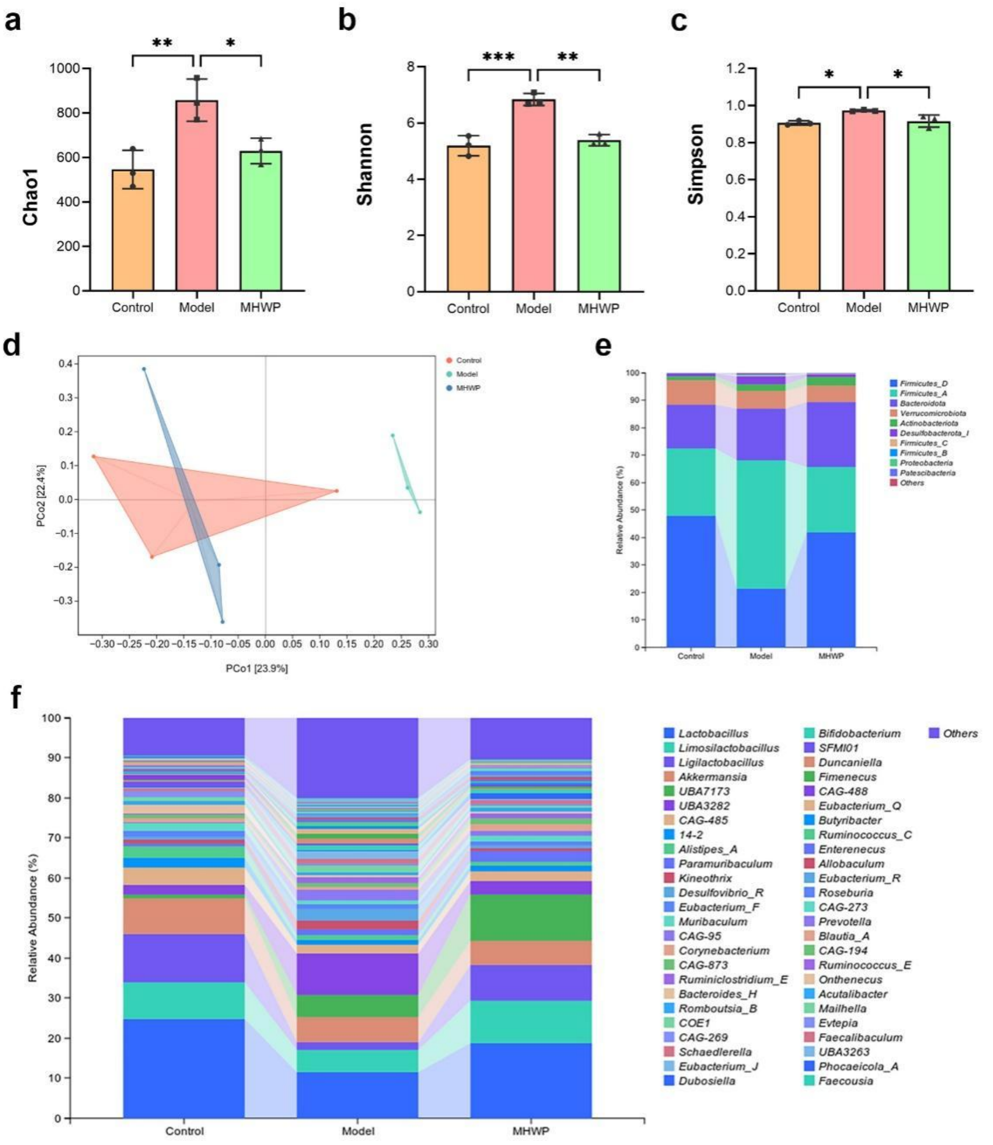

Supplementary Fig. S1 MHWP significantly altered ethanol-induced gut microbiota changes. a–c. α-Diversity analysis, encompassing the Chao1, Shannon, and Simpson indices, indicates significant shifts in microbial richness and diversity (n = 3). **d.** β-Diversity analysis (Bray–Curtis dissimilarity) reveals distinct clustering of microbial communities between treatment groups (n = 3). **e.** Taxonomic profiling at the phylum level. **f.** Taxonomic profiling at the Genus level.

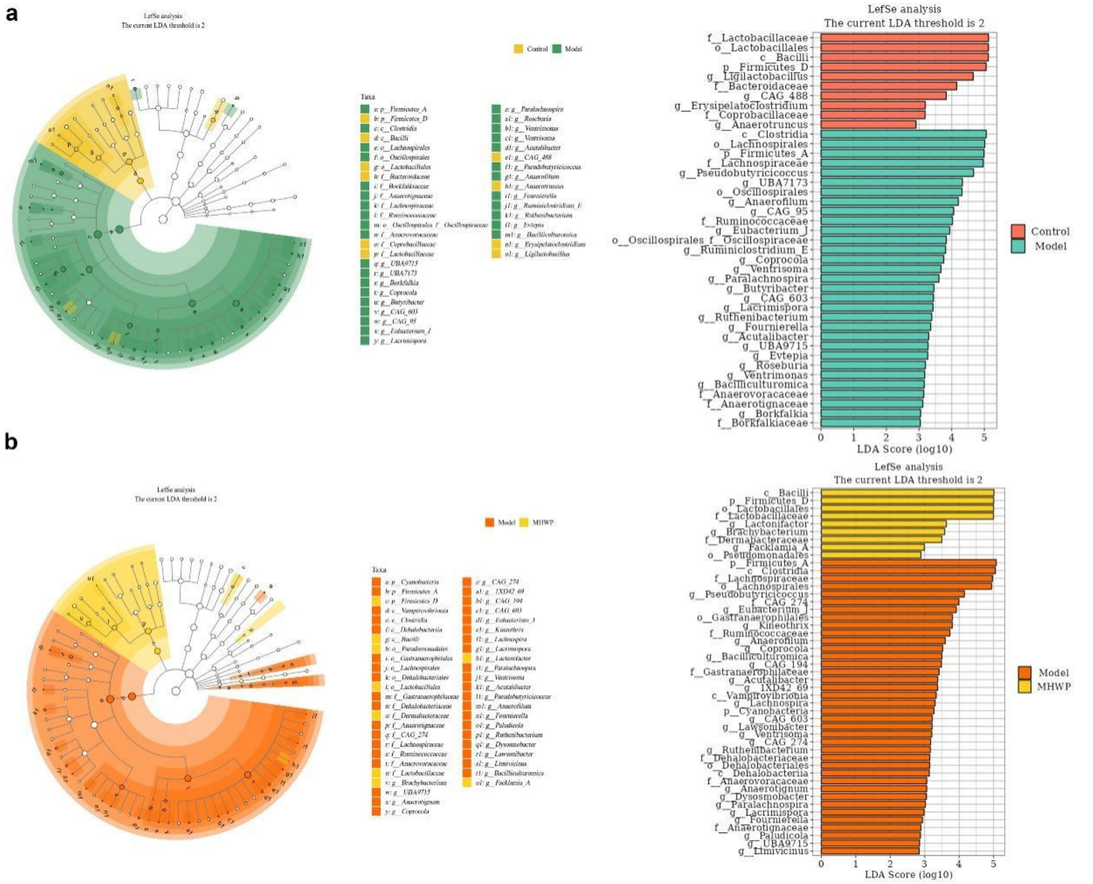

Supplementary Fig. S2 MHWP regulated the abundance of biomarkers of gut microbiota in ethanol-induced GMI rats. a. LDA value distribution histogram and cladogram of biomarkers between groups Control and Model. **b.** LDA value distribution histogram and cladogram of biomarkers between groups Model and MHWP. LDA score threshold >2 in a and b.

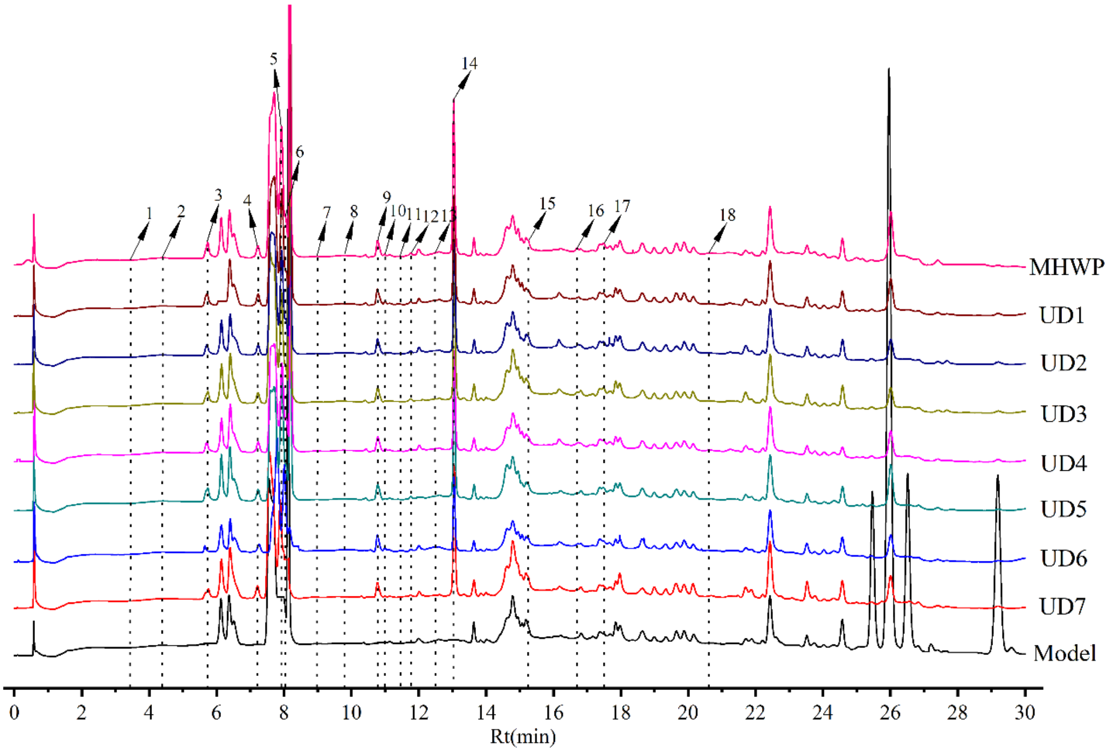

Supplementary Fig. S3. HPLC chromatograms of serum for each experimental group. The chromatograms illustrate the profiles for the MHWP group, the uniform design groups (UD1-UD7), and the Model control group. The arrows highlight the 18 distinct characteristic peaks identified after subtracting the shared peaks from the Model control group's profile.

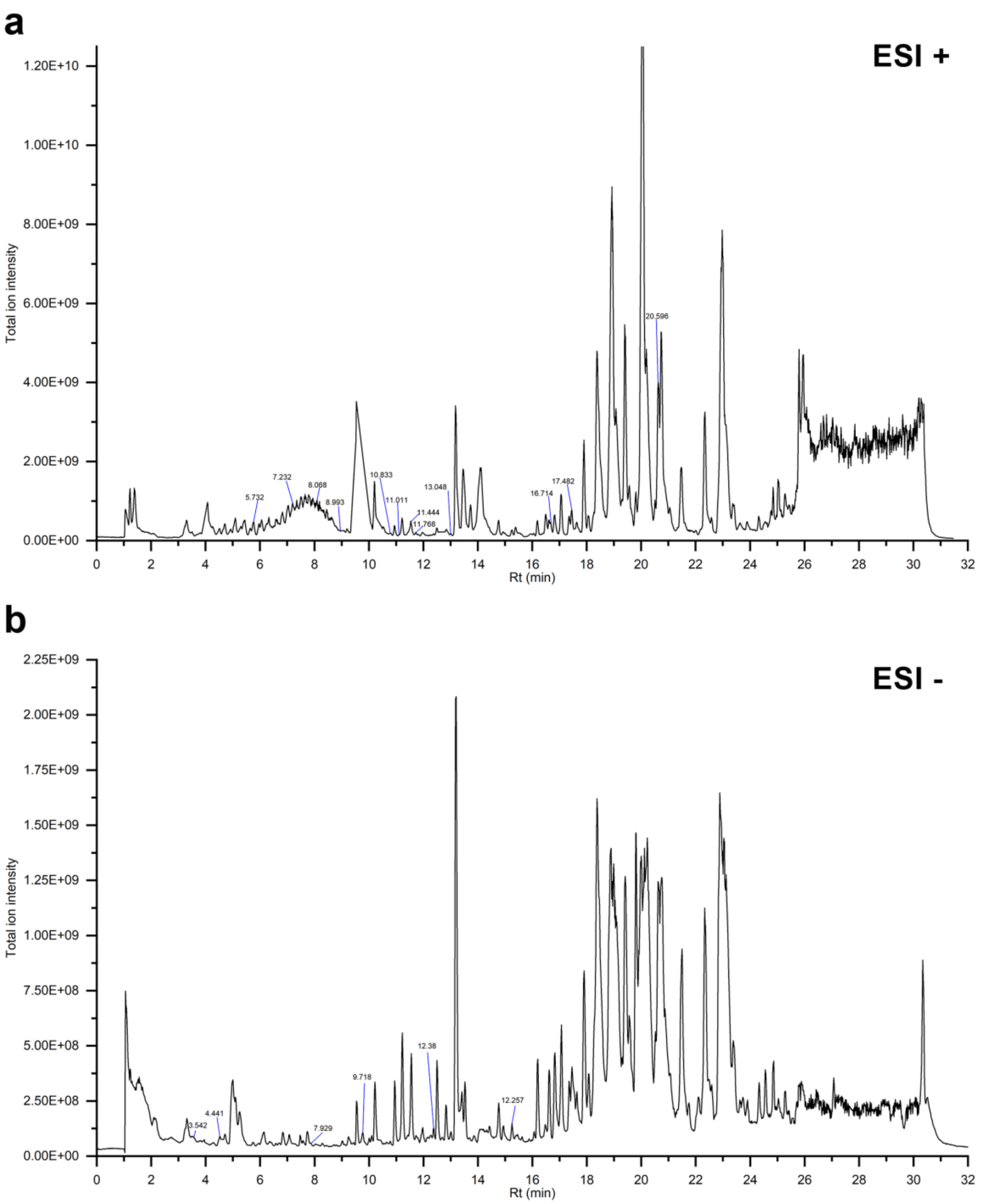

Supplementary Fig. S4. Total ion chromatograms (TIC) of MHWP-containing serum analyzed in both positive (a) and negative (b) ion modes using mass spectrometry (MS). The chromatograms show the retention times (Rt, min) and corresponding ion intensities, with the peaks highlighted in blue representing the specific plant-derived compounds in serum identified by comparison with the Model control group.
