# Supplementary material for: Gastric mucosal repair by Men’s Huwei Powder via EGF-NO/PGE2-PI3K-TLR4 in RELISH: Restoring Equilibrium through long-term integration of synergistic health
Source: Front Pharmacol. 2025 Jul 11;16:1594089. doi: 10.3389/fphar.2025.1594089 (PMC12289676; doi:10.3389/fphar.2025.1594089)
Supplement: Supplementary file 7 [file Presentation2.pdf]

The r code for PLSR analysis (Example: EGF)

1 Install and Load Required Packages

```
install.packages("readxl")
install.packages("pls")
install.packages('chillR')
install.packages('mdatools')
install.packages("writexl")

library(mdatools)
library(chillR)
library(readxl)
library(pls)
library(writexl)
```

2. Import Dataset for PLSR

```
file_SEM <- "C:/Users/73668/Desktop/Dataset used in PLSR&pSEM.xlsx"
SEM <- read_excel(file_SEM)
mode(SEM)
head(SEM)
```

3. PLSR Analysis of Gut Microbiota in Relation to the EGF-NO/PGE2 Axis

3.1 Define Predictor (x) and Response (y) Variables

```
#x: 50 microbiota genus (columns 105-122)
#y: Pharmacological indicator from the EGF-NO/PGE2 axis (EGF in column 31)
y<- plsr[31]
x<- plsr[55:104]
#Data Standardization and Type Conversion
x <- as.data.frame(lapply(x, function(col) {
  if (is.factor(col)) {
    as.numeric(as.character(col))
  } else if (is.character(col)) {
    as.numeric(col)
  } else {
    col
  }
}))
y <- as.data.frame(lapply(y, function(col) {
  if (is.factor(col)) {
    as.numeric(as.character(col))
  } else if (is.character(col)) {
    as.numeric(col)
  } else {
    col
  }
}))
```

3.2. Standardize Data

```
y<- scale(y)
x<- scale(x)
```

3.3. Cross-Validation for Optimal Component Selection (ncomp)

#Perform leave-one-out (LOO) cross-validation to evaluate pharmacological indicators in the EGF-NO/PGE2 axis (using EGF as an example) and determine the optimal number of components (ncomp) applicable to all indicators.

```
pls1<- plsr(y~x, validation="LOO", jackknife=TRUE)
summary(pls1)
```

3.4. Partial Least Squares Regression (PLSR) Analysis

```
# Model fitting with optimal number of components (ncomp = 1)
pls2<-plsr(y~x,validation="LOO",ncomp=1,jackknife=TRUE, method = "oscorespls")
summary(pls2)
# Model Performance Assessment: R-squared Calculation
pre_y = predict(pls2,ncomp = 1,newdata = x)
mse = sum((y-pre_y[,,1])**2/length(y))
R2 = 1-mse
print(R2)
# Extraction of Standardized Regression Coefficients
coefficients <- coef(pls2)
coefficients
# Variable Importance in Projection (VIP) Calculation
VIP <- VIP(pls2)
vip_df <- as.data.frame(VIP)
vip_df
```

4 PLSR Analysis of HPLC Fingerprints in Relation to the EGF-NO/PGE2 Axis

4.1. Define Predictor (x) and Response (y) Variables

```
#x: 18 characteristic peaks (columns 105-122)
#y: Pharmacological indicator from the EGF-NO/PGE2 axis (EGF in column 31)
y<- plsr[31]
x<- plsr[105:122]
# Data Standardization and Type Conversion
x <- as.data.frame(lapply(x, function(col) {
  if (is.factor(col)) {
    as.numeric(as.character(col))
  } else if (is.character(col)) {
    as.numeric(col)
  } else {
    col
  }
}))
y <- as.data.frame(lapply(y, function(col) {
  if (is.factor(col)) {
    as.numeric(as.character(col))
  } else if (is.character(col)) {
    as.numeric(col)
  } else {
    col
  }
}))
```

4.2 Standardize Data

```
y<- scale(y)
x<- scale(x)
```

4.3 Cross-Validation for Optimal Component Selection (ncomp)

```
#Perform leave-one-out (LOO) cross-validation to evaluate pharmacological indicators in the EGF-NO/PGE2 axis (using EGF as an example) and determine the optimal number of components (ncomp) applicable to all indicators.
pls1<- plsr(y~x, validation="LOO", jackknife=TRUE)
summary(pls1)
```

4.4 Partial Least Squares Regression (PLSR) Analysis

```
# Model fitting with optimal number of components (ncomp = 3)
pls2<-plsr(y~x,validation="LOO",ncomp=3,jackknife=TRUE, method = "oscorespls")
summary(pls2)
# Model Performance Assessment: R-squared Calculation
pre_y = predict(pls2,ncomp = 3,newdata = x)
mse = sum((y-pre_y[,,1])**2/length(y))
```

```
R2 = 1-mse
print(R2)
# Extraction of Standardized Regression Coefficients
coefficients <- coef(pls2)
coefficients
# Variable Importance in Projection (VIP) Calculation
VIP <- VIP(pls2)
vip_df <- as.data.frame(VIP)
vip_df
```
