# Supplementary material for: Gastric mucosal repair by Men’s Huwei Powder via EGF-NO/PGE2-PI3K-TLR4 in RELISH: Restoring Equilibrium through long-term integration of synergistic health
Source: Front Pharmacol. 2025 Jul 11;16:1594089. doi: 10.3389/fphar.2025.1594089 (PMC12289676; doi:10.3389/fphar.2025.1594089)
Supplement: Supplementary file 8 [file Presentation3.pdf]

The r code for pSEM

1. Install Required Packages

```
install.packages('piecewiseSEM')
install.packages("readxl")

library(readxl)
library(piecewiseSEM)
```

2. Data Import

```
file_path <- "C:/Users/73668/Desktop/Dataset used in LASSO&SPRA.xlsx"
pSEM <- read_excel(file_pSEM)
head(pSEM)
```

3. Fitting piecewise structural equation models

3.1 Fig.5

```
#Fig.5b
pSEM_Liver_MRS<-psem(
   lm(`NFκB.L` ~ ZR.ZR + ZR.PR, data = pSEM),
   lm(MRS ~ `NFκB.L`, data = pSEM),
   data = pSEM)
summary(pSEM_Liver_MRS)
fisherC(pSEM_Liver_MRS)
AIC_psem(pSEM_Liver_MRS)
#Fig.5c
pSEM_Liver_IL1β.G<-psem(
   lm(`NFκB.L` ~ LAR.ZR, data = pSEM),
   lm(IL1β.G ~ `NFκB.L`, data = pSEM),
   data = pSEM)
summary(pSEM_Liver_IL1β.G)
fisherC(pSEM_Liver_IL1β.G)
AIC_psem(pSEM_Liver_IL1β.G)
#Fig.5d
pSEM_Liver_IL6.G<-psem(
   lm(`TNFα.L` ~ ZR + ZR.GCR, data = pSEM),
   lm(IL6.G ~ `TNFα.L`, data = pSEM),
   data = pSEM)
summary(pSEM_Liver_IL6.G)
fisherC(pSEM_Liver_IL6.G)
AIC_psem(pSEM_Liver_IL6.G)
#Fig.5e
pSEM_Liver_IL1β.S<-psem(
   lm(`IL6.L` ~ ZR + CR.ZR, data = pSEM),
   lm(IL6.G ~ `IL6.L`, data = pSEM),
```

```
    data = pSEM)
summary(pSEM_Liver_IL1β.S)
fisherC(pSEM_Liver_IL1β.S)
AIC_psem(pSEM_Liver_IL1β.S)
#Fig.5f
pSEM_Liver_IL6.S<-psem(
    lm(`IL6.L` ~ ZR + ZR.PR, data = pSEM),
    lm(`NFκB.L` ~ ZR.PR + ZR.GCR, data = pSEM),
    lm(IL6.S ~ `NFκB.L` + `IL6.L`, data = pSEM),
    data = pSEM)
summary(pSEM_Liver_IL6.S)
fisherC(pSEM_Liver_IL6.S)
AIC_psem(pSEM_Liver_IL6.S)
#Fig.5g
pSEM_Liver_PI3K<-psem(
    lm(LF ~ ZR.ZR + CR, data = pSEM),
    lm(PI3K ~ LF, data = pSEM),
    data = pSEM)
summary(pSEM_Liver_PI3K)
fisherC(pSEM_Liver_PI3K)
AIC_psem(pSEM_Liver_PI3K)
#Fig.5h
pSEM_Liver_Akt<-psem(
    lm(`NFκB.L` ~ ZR.PR + ZR.GCR, data = pSEM),
    lm(Akt ~ `NFκB.L`, data = pSEM),
    data = pSEM)
summary(pSEM_Liver_Akt)
fisherC(pSEM_Liver_Akt)
AIC_psem(pSEM_Liver_Akt)
#Fig.5g
pSEM_Liver_NFκB.G<-psem(
    lm(`IL6.L` ~ ZR, data = pSEM),
    lm(`NFκB.G` ~ `IL6.L`, data = pSEM),
    data = pSEM)
summary(pSEM_Liver_NFκB.G)
fisherC(pSEM_Liver_NFκB.G)
AIC_psem(pSEM_Liver_NFκB.G)
```

3.2 Fig.6

```
#Fig.6b
pSEM_gut_MRS<-psem(
  lm(J13 ~ ZR.GCR, data = pSEM),
  lm(MRS ~ J13, data = pSEM),
  data = pSEM)
summary(pSEM_gut_MRS)
fisherC(pSEM_gut_MRS)
AIC_psem(pSEM_gut_MRS)
#Fig.6c
pSEM_gut_PGE2<-psem(
  lm(J13 ~ ZR.ZR + FF.PR + GCR, data = pSEM),
  lm(PGE2 ~ J13, data = pSEM),
  data = pSEM)
summary(pSEM_gut_PGE2)
fisherC(pSEM_gut_PGE2)
AIC_psem(pSEM_gut_PGE2)
#Fig.6d
pSEM_gut_COX2<-psem(
  lm(J43 ~ FF.PR + ZR.GCR, data = pSEM),
  lm(COX2 ~ J43, data = pSEM),
  data = pSEM)
summary(pSEM_gut_COX2)
fisherC(pSEM_gut_COX2)
AIC_psem(pSEM_gut_COX2)
#Fig.6e
pSEM_gut_RF<-psem(
  lm(J45 ~ LAR.FF, data = pSEM),
  lm(J13 ~ ZR.GCR, data = pSEM),
  lm(COX2 ~ J13 + J45, data = pSEM),
  data = pSEM)
summary(pSEM_gut_RF)
fisherC(pSEM_gut_RF)
AIC_psem(pSEM_gut_RF)
#Fig.6f
pSEM_gut_IL1β.G<-psem(
  lm(J38 ~ CR.PR, data = pSEM),
  lm(IL1β.G ~ J38, data = pSEM),
  data = pSEM)
summary(pSEM_gut_IL1β.G)
fisherC(pSEM_gut_IL1β.G)
AIC_psem(pSEM_gut_IL1β.G)
#Fig.6g
pSEM_gut_IL6.G<-psem(
```

```
    lm(J24 ~ LAR.ZR, data = pSEM),
    lm(J49 ~ ZR.GCR + LAR.LAR + FF, data = pSEM),
    lm(IL6.G ~ J24 + J49, data = pSEM),
    data = pSEM)
summary(pSEM_gut_IL6.G)
fisherC(pSEM_gut_IL6.G)
AIC_psem(pSEM_gut_IL6.G)
#Fig.6h
pSEM_gut_IL6.S<-psem(
    lm(J13 ~ ZR.GCR, data = pSEM),
    lm(J9 ~ ZR + LAR.FF, data = pSEM),
    lm(J45 ~ LAR.FF, data = pSEM),
    lm(IL6.S ~ J13 + J9 + J45, data = pSEM),
    data = pSEM)
summary(pSEM_gut_IL6.S)
fisherC(pSEM_gut_IL6.S)
AIC_psem(pSEM_gut_IL6.S)
#Fig.6i
pSEM_gut_PI3K<-psem(
    lm(J6 ~ GCR.GCR, data = pSEM),
    lm(PI3K ~ J6, data = pSEM),
    data = pSEM)
summary(pSEM_gut_PI3K)
fisherC(pSEM_gut_PI3K)
AIC_psem(pSEM_gut_PI3K)
#Fig.6j
pSEM_gut_Akt<-psem(
    lm(J12 ~ ZR.PR + LAR.ZR + ZR.GCR, data = pSEM),
    lm(J24 ~ LAR.ZR, data = pSEM),
    lm(Akt ~ J24 + J12, data = pSEM),
    data = pSEM)
summary(pSEM_gut_Akt)
fisherC(pSEM_gut_Akt)
AIC_psem(pSEM_gut_Akt)
#Fig.6k
pSEM_gut_NFκB.G<-psem(
    lm(J9 ~ ZR, data = pSEM),
    lm(J39 ~ PR.PR + CR.GCR + ZR.FF, data = pSEM),
    lm(J41 ~ ZR.FF, data = pSEM),
    lm(NFκB.G ~ J39 + J9 + J41, data = pSEM),
    data = pSEM)
summary(pSEM_gut_NFκB.G)
fisherC(pSEM_gut_NFκB.G)
AIC_psem(pSEM_gut_NFκB.G)
```

3.2 Fig.7

```
#Fig.7c
pSEM_fingerprint_MRS<-psem(
   lm(P1 ~ ZR.ZR, data = pSEM),
   lm(P10 ~ LAR.GCR, data = pSEM),
   lm(MRS ~ P1 + P10, data = pSEM),
   data = pSEM)
summary(pSEM_fingerprint_MRS)
fisherC(pSEM_fingerprint_MRS)
AIC_psem(pSEM_fingerprint_MRS)
#Fig.7d
pSEM_fingerprint_eNOS<-psem(
   lm(P18 ~ GCR.GCR, data = pSEM),
   lm(eNOS ~ P18, data = pSEM),
   data = pSEM)
summary(pSEM_fingerprint_eNOS)
fisherC(pSEM_fingerprint_eNOS)
AIC_psem(pSEM_fingerprint_eNOS)
#Fig.7e
pSEM_fingerprint_COX2<-psem(
   lm(P3 ~ LAR.ZR, data = pSEM),
   lm(P16 ~ ZR.GCR, data = pSEM),
   lm(COX2 ~ P3 + P16, data = pSEM),
   data = pSEM)
summary(pSEM_fingerprint_COX2)
fisherC(pSEM_fingerprint_COX2)
AIC_psem(pSEM_fingerprint_COX2)
#Fig.7f
pSEM_fingerprint_RF<-psem(
   lm(P7 ~ LAR.FF, data = pSEM),
   lm(RF ~ P7, data = pSEM),
   data = pSEM)
summary(pSEM_fingerprint_RF)
fisherC(pSEM_fingerprint_RF)
AIC_psem(pSEM_fingerprint_RF)
#Fig.7g
pSEM_fingerprint_IL1β.S<-psem(
   lm(P1 ~ ZR, data = pSEM),
   lm(IL1β.S ~ P1, data = pSEM),
   data = pSEM)
summary(pSEM_fingerprint_IL1β.S)
fisherC(pSEM_fingerprint_IL1β.S)
AIC_psem(pSEM_fingerprint_IL1β.S)
#Fig.7h
```

```
pSEM_fingerprint_IL6.S<-psem(
   lm(P11 ~ ZR.FF, data = pSEM),
   lm(IL6.S ~ P11, data = pSEM),
   data = pSEM)
summary(pSEM_fingerprint_IL6.S)
fisherC(pSEM_fingerprint_IL6.S)
AIC_psem(pSEM_fingerprint_IL6.S)
#Fig.7i
pSEM_fingerprint_TNFα.S<-psem(
   lm(P1 ~ ZR, data = pSEM),
   lm(TNFα.S ~ P1, data = pSEM),
   data = pSEM)
summary(pSEM_fingerprint_TNFα.S)
fisherC(pSEM_fingerprint_TNFα.S)
AIC_psem(pSEM_fingerprint_TNFα.S)
#Fig.7j
pSEM_fingerprint_IL1β.G<-psem(
   lm(P10 ~ LAR.ZR, data = pSEM),
   lm(IL1β.G ~ P10, data = pSEM),
   data = pSEM)
summary(pSEM_fingerprint_IL1β.G)
fisherC(pSEM_fingerprint_IL1β.G)
AIC_psem(pSEM_fingerprint_IL1β.G)
#Fig.7k
pSEM_fingerprint_IL6.G<-psem(
   lm(P3 ~ LAR.ZR, data = pSEM),
   lm(P16 ~ ZR.GCR, data = pSEM),
   lm(IL6.G ~ P3 + P16, data = pSEM),
   data = pSEM)
summary(pSEM_fingerprint_IL6.G)
fisherC(pSEM_fingerprint_IL6.G)
AIC_psem(pSEM_fingerprint_IL6.G)
#Fig.7l
pSEM_fingerprint_TNFα.G<-psem(
   lm(P8 ~ LAR.GCR, data = pSEM),
   lm(P10 ~ LAR.GCR, data = pSEM),
   lm(TNFα.G ~ P8 + P10, data = pSEM),
   data = pSEM)
summary(pSEM_fingerprint_TNFα.G)
fisherC(pSEM_fingerprint_TNFα.G)
AIC_psem(pSEM_fingerprint_TNFα.G)
#Fig.7m
pSEM_fingerprint_PI3K<-psem(
   lm(P18 ~ GCR.GCR, data = pSEM),
```

```
  lm(P10 ~ GCR.PR + ZR.ZR, data = pSEM),
  lm(PI3K ~ P18 + P10, data = pSEM),
  data = pSEM)
summary(pSEM_fingerprint_PI3K)
fisherC(pSEM_fingerprint_PI3K)
AIC_psem(pSEM_fingerprint_PI3K)
#Fig.7n
pSEM_fingerprint_Akt<-psem(
  lm(P3 ~ LAR.ZR, data = pSEM),
  lm(Akt ~ P3, data = pSEM),
  data = pSEM)
summary(pSEM_fingerprint_Akt)
fisherC(pSEM_fingerprint_Akt)
AIC_psem(pSEM_fingerprint_Akt)
#Fig.7o
pSEM_fingerprint_NFκB.G<-psem(
  lm(P1 ~ LAR.ZR, data = pSEM),
  lm(NFκB.G ~ P1, data = pSEM),
  data = pSEM)
summary(pSEM_fingerprint_NFκB.G)
fisherC(pSEM_fingerprint_NFκB.G)
AIC_psem(pSEM_fingerprint_NFκB.G)
```
